# Harnessing Medicinal Plants Through Advanced Drug Delivery: A New Era in Type 2 Diabetes Management

**DOI:** 10.3390/pharmaceutics18091100

**Published:** 2026-09-01

**Authors:** Abhishek Dadhich, Vikas Sharma, Shivika Sharma, Sweta Bawari, Iyyakkannu Sivanesan

**Affiliations:** 1 Department of Biotechnology, Graphic Era (Deemed to be University), Dehradun 248002, Uttarakhand, India; sabhi5061@gmail.com; 2 School of Bioengineering and Biosciences, Lovely Professional University, Jalandhar 144411, Punjab, India; biotech_vikas@rediffmail.com (V.S.);; 3 Amity Institute of Pharmacy, Amity University Campus, Sector-125, Noida 201301, Uttar Pradesh, India; vivacious.sweta@gmail.com; 4 Department of Environmental Health Science, Human and Eco Care Center, Konkuk University, Hwayang-dong, Gwangjin-gu, Seoul 05029, Republic of Korea

**Keywords:** phytochemicals, nanocarriers, oral bioavailability, curcumin, berberine, AMPK, NF-κB, lipid nanoparticles, nano-phytomedicine, gut microbiota, translational pharmacology

## Abstract

Type 2 diabetes mellitus (T2DM) remains a global health crisis, with nearly 589 million adults currently affected and projections pointing toward 853 million by 2050. Despite an expanding pharmacological armamentarium, a significant proportion of patients fail to achieve adequate glycaemic control, and the limitations of existing therapies, including adverse effects, cost, and limited accessibility, underscore the compelling need for novel therapeutic approaches. Phytochemicals such as curcumin, berberine, quercetin, resveratrol, and epigallocatechin gallate possess well-documented antidiabetic activity, operating through the PI3K/Akt, AMPK (activated protein kinase), NF-κB/JNK (nuclear factor kappa-B), and GLP-1R (glucagon-like peptide-1) signalling axes to improve insulin sensitivity, suppress gluconeogenesis, protect pancreatic beta-cells, and attenuate chronic metabolic inflammation. However, their clinical utility has been fundamentally constrained by poor oral bioavailability arising from low aqueous solubility, gastrointestinal instability, extensive first-pass metabolism, and P-glycoprotein-mediated efflux. Advanced drug delivery systems, including liposomes, solid lipid nanoparticles (SLN), nanostructured lipid carriers, PLGA (Poly (lactic-co-glycolic acid)) and chitosan nanoparticles, nanoemulsions, self-nanoemulsifying drug delivery systems, and phytosomes have demonstrated the capacity to overcome these barriers, achieving five- to ten-fold improvements in systemic bioavailability and substantially enhanced antidiabetic efficacy in preclinical models. Emerging mechanistic evidence further positions gut microbiota modulation and epigenetic reprogramming as additional therapeutic axes through which nano-encapsulated phytochemicals may exert durable metabolic benefits. Nonetheless, critical translational challenges persist, encompassing nanotoxicological risks, herb–drug interactions, the absence of harmonised regulatory frameworks for nano-phytomedicine products, phytochemical raw material variability, and formidable technical and economic barriers to scalable nanoparticle manufacturing. This review synthesises the current mechanistic, formulation, and clinical evidence within a unified analytical framework and identifies the strategic priorities of rigorous clinical development, regulatory clarity, and manufacturing standardisation required to translate nano-phytomedicine science into evidence-based T2DM therapeutics.

## 1. Introduction

Diabetes mellitus is one of the greatest epidemics of chronic disease in the twenty-first century, and more than 90% of all diagnosed cases are attributable to type 2 diabetes mellitus (T2DM). The 11th edition of the International Diabetes Federation (IDF) Diabetes Atlas (2024) estimates that 589 million adults currently live with diabetes, representing 11.1% of the global adult population, with projections that 853 million will be affected by 2050 [1]. The human cost has been profound: the disease claims an estimated 3.4 million lives annually and imposes more than USD 1 trillion in direct health expenditure. Critically, approximately 43% of those affected remain undiagnosed, and the overwhelming majority reside in low- and middle-income countries whose health systems are least equipped to manage a condition of such chronicity and complexity [1].

T2DM is characterised by two closely interacting pathological events: progressive peripheral insulin resistance and a concurrent decline in the insulin secretory capacity of pancreatic β-cells. Inadequately managed, the resulting chronic hyperglycaemia precipitates microvascular and macrovascular complications, cardiovascular disease, diabetic nephropathy, peripheral neuropathy, and diabetic retinopathy that substantially reduce quality of life and life expectancy [2]. Although the pharmacological armamentarium for T2DM has expanded considerably, a large proportion of patients fail to achieve or sustain adequate glycaemic control [2]. First-line agents such as metformin are generally well tolerated but carry risks of gastrointestinal adverse effects and lactic acidosis in patients with renal impairment, and their efficacy diminishes as β-cell mass declines. Glucagon-like peptide 1 (GLP-1) receptor agonists and sodium–glucose cotransporter-2 (SGLT-2) inhibitors represent genuine therapeutic advances but remain inaccessible in resource-limited settings; sulfonylureas risk hypoglycaemia; and thiazolidinediones cause weight gain. Collectively, these limitations sustain a substantial unmet therapeutic need and have maintained enduring scientific interest in plant-based alternatives [3].

The management of diabetes has deep roots in medicinal plant traditions across diverse healing systems, and several foundational synthetic drugs were themselves plant-derived: metformin originates from galegine, the active alkaloid of *Galega officinalis* [3,4]. This ethnopharmacological heritage is now underpinned by mechanistic studies demonstrating that phytochemicals including curcumin, berberine, quercetin, resveratrol, gymnemic acid, and andrographolide exert substantial antidiabetic effects through structurally distinct mechanisms. These compounds reduce insulin resistance via the phosphoinositide-3-kinase (PI3K)/v-akt murine thymoma viral oncogene homologue (AKT) and AMP-activated protein kinase (AMPK) signalling pathways, suppress chronic low-grade inflammation through inhibition of nuclear factor kappa-light-chain-enhancer of activated B cells (NF-κB), preserve β-cell mass from glucotoxic and lipotoxic stress, and inhibit intestinal α-glucosidase to blunt postprandial glucose excursions [5,6]. Importantly, the capacity to engage multiple pathogenic pathways simultaneously aligns naturally with the multifactorial pathobiology of T2DM in a manner that single-target synthetic agents typically cannot replicate [5,7].

However, these promising compounds have been consistently constrained by a fundamental biopharmaceutical challenge: inadequate oral bioavailability. Antidiabetic phytochemicals suffer from limited aqueous solubility, rapid degradation in the gastrointestinal tract, extensive first-pass hepatic metabolism, and active efflux from intestinal epithelial cells mediated by P-glycoprotein [8,9]. Curcumin, perhaps the most extensively studied dietary polyphenol, exhibits less than 1% oral bioavailability in conventional formulations, attributable largely to its aqueous insolubility (~11 ng/mL) and susceptibility to phase II conjugation [9,10]. The combined effect is a pharmacokinetic profile characterised by low systemic exposure, high inter-individual variability, and plasma concentrations well below those required to reproduce the antidiabetic effects observed in preclinical systems, limiting phytochemicals to supplementary rather than primary therapeutic roles despite robust mechanistic justification. Advanced drug delivery technologies have emerged as a compelling strategy to overcome these restrictions [11,12]. Enclosing bioactive phytochemicals within nanocarrier architectures including liposomes, solid lipid nanoparticles (SLNs), nanostructured lipid carriers (NLCs), poly(lactide-co-glycolide) (PLGA) nanoparticles, chitosan nanoparticles, nanoemulsions, phytosomes, and self-nanoemulsifying drug delivery systems (SNEDDS) protects the payload from premature degradation, circumvents biological barriers to absorption, prolongs systemic circulation, and enables targeted delivery to metabolically relevant tissues [13,14]. Preclinical studies demonstrate that such formulations can achieve five- to ten-fold improvements in systemic bioavailability alongside substantially enhanced antidiabetic efficacy: curcumin-loaded SLNs have shown up to 9.5-fold improvement in oral bioavailability in rodent pharmacokinetic studies, while berberine-loaded SNEDDS formulations have increased plasma exposure by approximately 3.2-fold compared with conventional dosage forms [15,16,17,18]. The integration of phytochemical pharmacology with nanotechnology-enabled delivery thus represents not merely an incremental formulation advance but a strategic repositioning of plant-derived therapeutics as viable, mechanism-driven candidates for evidence-based T2DM management [14].

This review takes a unified analytical approach spanning these two interconnected and rapidly evolving fields. It begins with a description of the molecular pathophysiology of T2DM and the key signalling nodes amenable to phytochemical intervention. It then critically examines the major classes of antidiabetic phytochemicals polyphenols, alkaloids, terpenoids, saponins, and dietary fibre fractions with respect to their chemistry, mechanisms, and available preclinical and clinical evidence. The central section surveys the structural design and encapsulation performance of nanocarrier platforms for phytochemical delivery, with emphasis on in vivo antidiabetic outcomes. Additional mechanistic dimensions, including gut microbiota modulation and epigenetic reprogramming, are incorporated. Finally, the review addresses the toxicological, regulatory, and manufacturing challenges that must be resolved to translate nano-phytomedicine science into clinical reality. Although related mechanistic surveys including a recently published review covering phytochemical signalling pathways, bibliometric trends, structure–activity relationships, and cAMP/PKA (cyclic adenosine monophosphate/protein kinase A) and MAPK (mitogen-activated protein kinase) pathway diagrams in T2DM address the biological landscape of this field comprehensively, the present manuscript is distinguished by its evidence quality appraisal architecture: a GRADE (grading of recommendations assessment, development and evaluation)-rated clinical evidence framework, a five-category structured evidence table with verified primary-source citations, a pharmacokinetic herb–drug interaction analysis, a head-to-head nanocarrier platform comparison across ten parameters, and a systematic regulatory pathway analysis covering FDA (Food and Drug Administration), EMA (European Medicines Agency), and CDSCO (Central Drugs Standard Control Organisation) frameworks. The literature base spans peer-reviewed publications primarily from 2015 to 2026, drawn from PubMed, Scopus, and Web of Science, and is intended to serve both researchers working at the natural products–nanomedicine interface and those concerned with the translational priorities for evidence-based metabolic disease therapeutics.

## 2. Scope, Novelty, and Literature Search Strategy

### 2.1. Differentiation from Prior Reviews

Several reviews have addressed phytochemicals in T2DM and nanoformulation strategies individually; however, no single review has comprehensively integrated mechanistic evidence, nanocarrier platform comparisons, structured clinical evidence grading, herb–drug interaction profiling, and regulatory pathway analysis within a single unified framework targeting T2DM. Table S1 compares the scope and contribution of the present manuscript with five representative prior reviews identified during the search process.

### 2.2. Literature Search Strategy and Study Selection

A systematic literature search was conducted across four electronic databases: PubMed/MEDLINE, Scopus, Web of Science, and Google Scholar, covering January 2000 to August 2025, with particular emphasis on publications from 2018 onwards to reflect contemporary advances in nanoformulation science and antidiabetic phytochemical research. Search terms were constructed using Boolean operators combining Medical Subject Headings (MeSH) and free-text keywords across three domains. Domain 1 (Disease): “type 2 diabetes mellitus” OR “T2DM” OR “insulin resistance” OR “hyperglycaemia” OR “prediabetes.” Domain 2 (Phytochemicals): “curcumin” OR “berberine” OR “quercetin” OR “resveratrol” OR “epigallocatechin gallate” OR “EGCG (epigallocatechin gallate)” OR “andrographolide” OR “ginsenoside” OR “gymnemic acid” OR “cinnamaldehyde” OR “medicinal plant” OR “phytochemical” OR “polyphenol.” Domain 3 (Nanoformulation): “nanoparticle” OR “nanocarrier” OR “solid lipid nanoparticle” OR “SLN” OR “PLGA” OR “chitosan nanoparticle” OR “nanoemulsion” OR “SNEDDS” OR “phytosome” OR “liposome” OR “drug delivery” OR “bioavailability.” Domain combinations searched were D1 AND D2; D1 AND D3; and D1 AND D2 AND D3.

Inclusion criteria encompassed English-language publications; original research articles, systematic reviews, meta-analyses, and clinical trials; in vitro mechanistic studies with clearly defined experimental models; in vivo preclinical studies using validated diabetic animal models including streptozotocin-induced, db/db, ob/ob, and high-fat-diet-fed rodent models; human clinical trials including randomised controlled trials, open-label studies, and pilot studies with defined T2DM or prediabetes populations; and studies reporting quantitative outcomes such as fasting blood glucose, HbA1c, HOMA-IR, oral bioavailability parameters, or minimum physicochemical nanoparticle characterisation data including particle size, zeta potential, and encapsulation efficiency.

Exclusion criteria encompassed non-English publications; conference abstracts, editorials, and opinion pieces without original data; studies with insufficient methodological detail for quality assessment; studies addressing Type 1 diabetes mellitus exclusively; nanoformulation studies not reporting minimum physicochemical characterisation; clinical studies without trial registration or a verifiable primary source with DOI; and studies reporting specific numerical parameters sample sizes, durations, or endpoints without a citable primary source traceable to a registered trial or peer-reviewed publication with DOI.

**Figure 1:**
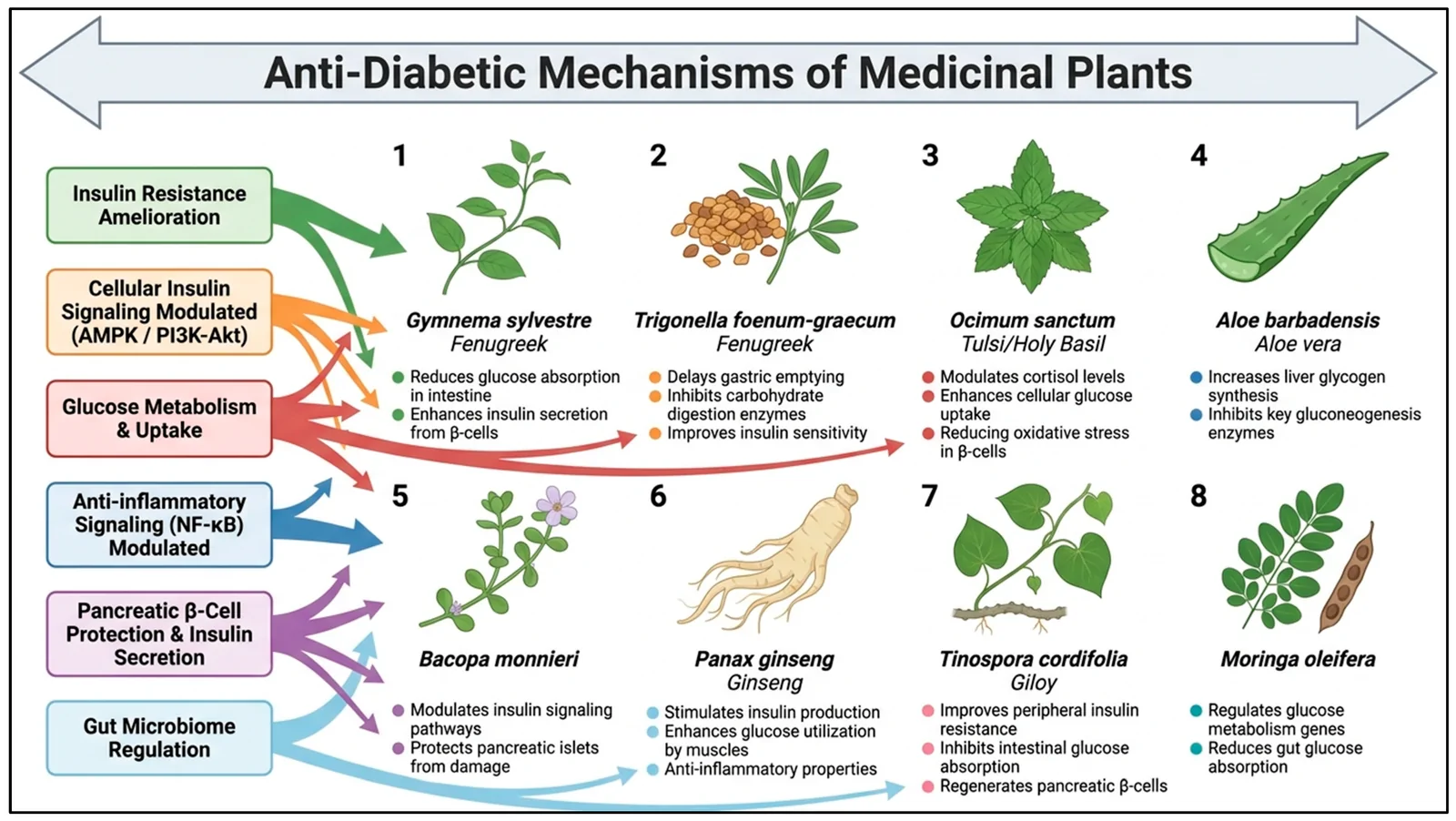
Medicinal plants and their mechanisms in managing insulin resistance and glucose metabolism.

**Figure 2:**
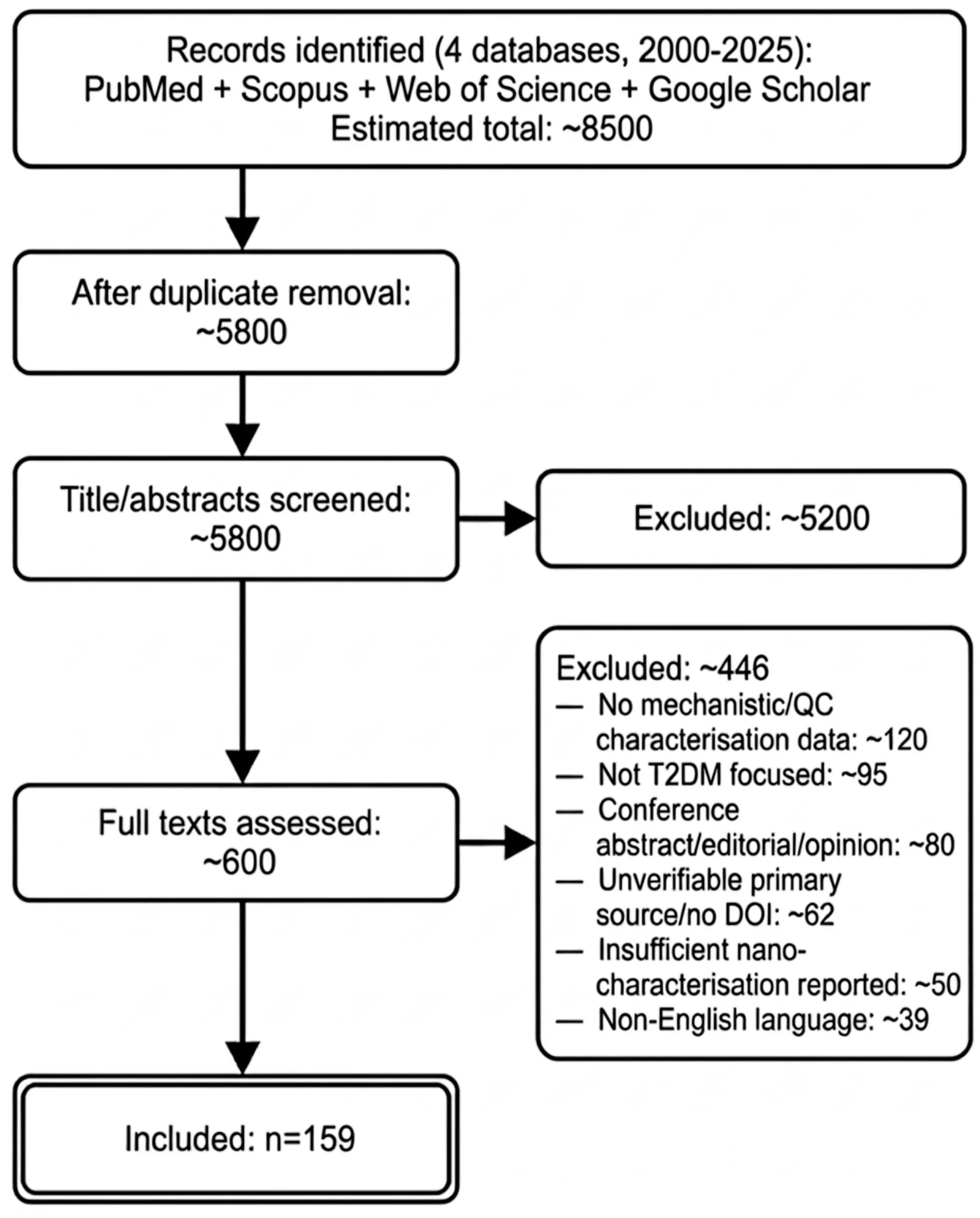
PRISMA flow diagram of the systematic literature search and study selection process.

Study quality assessment was performed using a three-tier framework. Human RCTs and meta-analyses of RCTs were rated using the GRADE framework, with per-phytochemical ratings. Preclinical in vivo studies were assessed for model validity, presence of an appropriate diabetic induction method, randomisation, blinding, and outcome reporting completeness. In vitro mechanistic studies were assessed for physiological relevance of compound concentrations relative to achievable in vivo exposures, cell line appropriateness, and reproducibility of reported mechanistic data. All citations included in evidence tables (Tables 1–5) were individually verified by PubMed PMID confirmation and DOI validation prior to inclusion. The study selection process is summarised in Figure 1 (PRISMA (Preferred Reporting Items for Systematic Reviews and Meta-Analyses) flow diagram). Following database searching, titles and abstracts were independently screened against inclusion and exclusion criteria; full texts of potentially eligible studies were subsequently retrieved and assessed. Reference lists of included systematic reviews and meta-analyses were hand-searched to identify additional eligible primary studies not captured by the database search (Figure 2).

## 3. Pathophysiology of Type 2 Diabetes: Molecular Targets Amenable to Phytotherapy

A meaningful evaluation of phytochemical strategies in T2DM demands clarity on the molecular pathology these strategies address. T2DM is a systemic metabolic disorder in which insulin resistance and β-cell failure unfold across skeletal muscle, liver, adipose tissue, and the endocrine pancreas [19,20]. Understanding the signalling nodes that sustain this state is essential for appreciating the mechanistic rationale behind phytotherapy and for identifying where nanocarrier delivery yields the greatest benefit.

### 3.1. Insulin Resistance, β-Cell Dysfunction, and Glucotoxicity: Core Mechanistic Triad

Insulin resistance manifests principally as a defect in GLUT4 (glucose transporter type 4) vesicle trafficking to the plasma membrane in skeletal muscle, which normally accounts for 70–80% of postprandial glucose disposal [20,21]. Intracellular lipid intermediates, diacylglycerols and ceramides, drive serine phosphorylation of IRS-1 (insulin receptor substrate-1), inhibiting the tyrosine phosphorylation required for PI3K activation and downstream Akt-mediated GLUT4 translocation [20]. In the liver, impaired suppression of gluconeogenesis despite hyperinsulinemia creates a self-reinforcing cycle of fasting hyperglycaemia [19,20].

Progressive loss of β-cell mass and function is driven by glucotoxicity, lipotoxicity, ER stress, and islet inflammation [22]. Glucotoxicity reduces expression of PDX-1 (pancreatic and duodenal homeobox factor 1), NKX6.1 (NK6 transcription factor-related, locus 1), and MafA (v-maf musculoaponeurotic fibrosarcoma oncogene homologue A), impairing glucose-stimulated insulin secretion and promoting apoptosis. Excess ROS generated via the polyol, hexosamine, PKC, and AGE pathways underlie oxidative β-cell injury [23]. Concurrent lipotoxicity, driven by elevated free fatty acids, triggers ceramide accumulation and ER stress via the unfolded protein response, compounding β-cell damage [22,24,25]. The simultaneous operation of these stressors creates glucolipotoxicity, now recognised as a primary cause of β-cell failure [22,24]. Shared mediators of chronic inflammation and oxidative stress link all three pathological processes and are amenable to phytochemical intervention [6,7,26].

### 3.2. Key Signalling Axes (PI3K/Akt, AMPK, NF-κB/JNK, GLP-1R) as Druggable Nodes

PI3K/Akt: Insulin binding activates PI3K → PIP3 (phosphatidylinositol (3,4,5)-trisphosphate) → Akt (phosphorylated at Thr308/Ser473), enabling AS160-mediated GLUT4 translocation and glycogen synthase kinase-3 beta (GSK-3β) inhibition, promoting glycogen synthesis [27,28,29,30]. Phytochemicals including curcumin, quercetin, berberine, and anthocyanins restore IRS-1 tyrosine phosphorylation and downstream Akt activation in insulin-resistant models, thereby enhancing GLUT4 translocation and glucose uptake [29,30]. Williamson and Sheedy (2020) systematically reviewed the evidence that quercetin, epicatechin, and anthocyanins modulate insulin signalling through PI3K/Akt/GLUT4, with supporting evidence from human intervention studies [29].

AMPK (activated by Thr172 phosphorylation when AMP (adenosine monophosphate)/ATP (adenosine triphosphate) rises) increases glucose uptake, promotes fatty acid oxidation, and suppresses hepatic gluconeogenesis via PEPCK (phosphoenolpyruvate carboxykinase)/G6Pase (glucose-6-phosphatase) downregulation, which is analogous to metformin’s primary action [27,31,32,33]. Berberine is the most extensively studied phytochemical AMPK activator [34]. Through mild complex I inhibition (raising AMP/ATP), it reduces hepatic gluconeogenesis and enhances GLUT4-mediated skeletal muscle glucose uptake [27,35]. The structural basis of AMPK modulation by small molecules has been comprehensively reviewed [28]. NF-κB/JNK. Pro-inflammatory cytokines (TNF-α (tumour necrosis factor-alpha), IL-1β, IL-6 (interleukin-6)) activate IKKβ (inhibitor of nuclear factor kappa-B kinase subunit beta), triggering NF-κB p65/p50 nuclear translocation and transcription of further inflammatory mediators. JNK concurrently phosphorylates IRS-1 at inhibitory serine residues, sustaining insulin resistance [20,31]. Curcumin interrupts this at multiple points: it inhibits IKKβ activation, suppresses NF-κB nuclear translocation, and attenuates ERK/JNK phosphorylation in hyperglycaemic insulin-resistant cells, restoring PI3K/Akt/GSK3β signalling [33,34]. Berberine similarly suppresses NF-κB-driven inflammatory cascades through AMPK-dependent and -independent mechanisms, reducing cytokine-mediated impairment of IRS-1 phosphorylation [35].

GLP-1R axis: GLP-1, secreted from intestinal L-cells, enhances glucose-driven insulin secretion, suppresses glucagon, and exerts β-cell trophic effects; this incretin action is substantially blunted in T2DM. Certain phytochemicals engage this axis indirectly: *Gymnema sylvestre* extracts stimulate GLP-1 release from L-cells and promote β-cell regeneration; quercetin both stimulates GLP-1 secretion and inhibits DPP-4 (dipeptidyl peptidase-4), extending incretin activity [5,36,37]. Myricetin inhibits DPP-4 and elevates circulating GLP-1 levels in streptozotocin-induced diabetic rats, improving GSIS (glucose-stimulated insulin secretion) and reducing DPP-4/NLRP3 (nucleotide-binding oligomerisation domain, leucine-rich repeat and pyrin domain-containing 3) inflammasome activation [36]. These findings identify phytochemical modulation of the GLP-1R axis as an underexplored strategy requiring sustained intestinal delivery that nanocarrier platforms can provide [38].

## 4. Antidiabetic Phytochemicals: Chemistry, Mechanisms, and Evidence

The antidiabetic phytochemicals discussed in this review are drawn from five structural families: curcuminoids, flavonoids, stilbenoids, alkaloids, and terpenoid/saponin-rich fractions. Table 1 provides a standardised taxonomy by chemical class alongside key mechanisms and evidence. This section focuses on the mechanistic depth and translational context of each class. Throughout Section 4, evidence statements are labelled by study type to enable unambiguous interpretation: [IV] = in vitro (cell-based or enzymatic assay); [PC] = preclinical in vivo (animal model); [CC] = clinical-conventional (human trial of non-nanoformulated preparation, presented as mechanistic benchmark only); [CN] = clinical-nano (human trial of verified nanoformulated preparation). [CC] data confirm target engagement and therapeutic direction but do not constitute evidence that nanoformulations will achieve equivalent or superior clinical outcomes in T2DM.

### 4.1. Polyphenols

#### 4.1.1. Curcumin

Curcumin (1,7-bis(4-hydroxy-3-methoxyphenyl)-1,6-heptadiene-3,5-dione) is the principal curcuminoid of *Curcuma longa* rhizome, constituting 77% of the total curcuminoid fraction alongside demethoxycurcumin and bisdemethoxycurcumin. Its β-diketone tautomerism, phenolic hydroxyl groups, and Michael acceptor α,β-unsaturated carbonyl confer broad polypharmacological reactivity, enabling covalent modification of key cysteine residues in NF-κB and Keap1 [10,39]. Curcumin inhibits NF-κB and suppresses IL-6/TNF-α in macrophage and β-cell lines at 10–50 μmol/L. [PC] Oral curcumin 100–200 mg/kg reduces FBG and preserves islet architecture in STZ-induced diabetic rodents. [CC] In human trials of conventional curcumin preparations, HbA1c ↓0.32% and FBG ↓4.60 mg/dL across 59 RCTs. [CN] No true nanoparticle curcumin RCT in T2DM has been registered; phytosomal Meriva^®^ (phospholipid complex, not a nanoparticle) represents the most advanced formulation-enhanced clinical evidence [33,40,41].

#### 4.1.2. Quercetin

Quercetin (3,3′,4′,5,7-pentahydroxyflavone), a flavonol ubiquitous in onions, capers, apples, and tea, exhibits multi-target antidiabetic activity. [IV] Quercetin competitively inhibits α-glucosidase with IC_50_ (half-maximal inhibitory concentration) values comparable to acarbose in isolated enzyme assays. [PC] Quercetin nanoemulsion (125 nm) reduces FBG and preserves hepatocyte histology in STZ diabetic rats. [CC] Umbrella meta-analysis of 18 RCTs (conventional quercetin) shows insulin WMD −1.07 μIU/mL; FBG not significantly changed. [CN] No nano-quercetin human RCT identified [29,42]. Quercetin activates SIRT1 (sirtuin-1 (SIRT1), deacetylating key metabolic regulators and improving insulin sensitivity. Clinical evidence for quercetin as an isolated compound remains limited, but combined interventions and nanoemulsion formulations show improvements in fasting glucose and lipid profiles in small studies [43].

#### 4.1.3. Resveratrol

[IV] Resveratrol activates SIRT1 and AMPK in hepatocyte and adipocyte cell lines at 10–50 μmol/L. [PC] SLN-encapsulated resveratrol improves bioavailability 3–5-fold and reduces FBG in preclinical models. [CC] Small-scale human RCTs (n = 19–75) of conventional resveratrol show highly variable glycaemic outcomes. [CN] No nano-resveratrol RCT in T2DM exists; all nanoformulation data are preclinical only [44,45,46,47]. It reduces hepatic gluconeogenesis, improves endothelial nitric oxide bioavailability, and attenuates mitochondrial oxidative stress in diabetic rodent models [44]. Chitosan-stabilised resveratrol/selenium nanoparticles demonstrate enhanced antidiabetic efficacy versus free resveratrol in streptozotocin/high-fat diet mouse models, reducing fasting glucose, improving insulin resistance indices, and attenuating inflammatory cytokines via PI3K/Akt/mTOR engagement [46].

#### 4.1.4. Epigallocatechin-3-Gallate (EGCG)

EGCG, the most abundant flavan-3-ol catechin of *Camellia sinensis* green tea, targets multiple pathophysiological nodes of T2DM, the so-called “ominous octet”, including β-cell dysfunction, hepatic insulin resistance, gut dysbiosis, and glucotoxicity [48,49]. EGCG inhibits α-glucosidase, DPP-4, and sodium-glucose transport proteins, reducing intestinal glucose absorption and renal glucose reabsorption. It activates AMPK and PI3K/Akt in skeletal muscle, restores gut microbiota composition by enriching *Akkermansia muciniphila* and *Lactobacillus* species, and reduces intestinal permeability, thereby reducing metabolic endotoxaemia that aggravates insulin resistance [49]. Its principal pharmacokinetic limitation is instability at intestinal pH and susceptibility to O-methylation and glucuronidation, reducing systemic exposure after oral ingestion [8]. [Epidemiological] Tea consumption ≥ 4 cups/day is associated with a 17% reduced T2DM risk across 19 cohort studies (n > 1,076,311); this reflects dietary green tea, not isolated EGCG or nanoformulation. [IV] EGCG competitively inhibits α-glucosidase and activates AMPK in cell-based assays. [CN] No nano-EGCG human RCT in T2DM identified.

### 4.2. Alkaloids

#### 4.2.1. Berberine

Berberine, an isoquinoline alkaloid (protoberberine type) isolated from *Berberis aristata*, *Coptis chinensis*, and *Phellodendron amurense*, is the most extensively characterised plant-derived antidiabetic alkaloid. Its mechanisms closely parallel those of metformin: mild complex I inhibition raises the intracellular AMP/ATP ratio, activating AMPK and downstream suppression of hepatic gluconeogenesis (via PEPCK and G6Pase downregulation), upregulation of GLUT1 and GLUT4 in skeletal muscle, and inhibition of lipogenesis via acetyl-CoA carboxylase [27,34]. Berberine also suppresses NF-κB-mediated inflammatory cascades, reducing pro-inflammatory cytokine expression that impairs IRS-1 tyrosine phosphorylation [34]. [IV] Berberine activates AMPK via Complex I inhibition in hepatocytes, skeletal muscle cells, and adipocytes at 1–10 μmol/L. [PC] Oral berberine 50–200 mg/kg reduces FBG and improves lipid profiles in HFD/STZ rodent models. [CC] Berberine 500 mg × 3/day (conventional extract) reduces FBG by 22–36% and HbA1c by 0.9–1.2% in Chinese T2DM RCTs, with effects comparable to metformin. [CN] No nano-berberine RCT exists; chitosan-coated SLN berberine data are preclinical only [16].

#### 4.2.2. Andrographolide

Andrographolide, a bicyclic diterpenoid lactone (labdane-type) from *Andrographis paniculata*, targets NF-κB and the PI3K/Akt/GSK-3β axis. Its α-methylene-γ-butyrolactone moiety forms a covalent adduct with the p50 subunit of NF-κB, irreversibly blocking nuclear translocation and downstream inflammatory cytokine transcription; this disruption of the NF-κB/IRS-1 serine phosphorylation cycle restores insulin signal transduction in metabolic tissues [50]. In STZ-induced diabetic rodents, andrographolide supplementation significantly reduces fasting blood glucose, improves glucose tolerance, and exerts protective effects on pancreatic islet morphology, attributed to partial restoration of β-cell mass and reduced oxidative injury [51]. Clinical data for nanoformulated andrographolide are not yet available.

### 4.3. Terpenoids, Saponins, and Dietary-Fibre-Rich Fractions

#### 4.3.1. Gymnemic Acids (*Gymnema sylvestre*)

Gymnemic acid, a soleanane-type triterpenoid saponin based on the aglycone oleanolic acid, constitutes the principal bioactive fraction of *Gymnema sylvestre* (Gurmar). They inhibit intestinal glucose absorption by blocking glucose-binding sites in the intestinal brush border, suppress sweet-taste receptor activation (reducing sucrose palatability and cephalic-phase insulin response), stimulate GLP-1 secretion from L-cells, and promote β-cell regeneration in experimental diabetic models [48,50]. A systematic review of clinical studies demonstrates reductions in fasting glucose and postprandial glucose in patients with T2DM, with improvements in HbA1c and body weight reported in several trials of aqueous leaf extracts [52].

#### 4.3.2. Ginsenosides (*Panax ginseng*)

Ginsenosides, dammarane-type tetracyclic triterpenoid saponins, constitute the principal bioactive fraction of *Panax ginseng* and related species. More than 100 structurally distinct ginsenosides have been identified, classified by sugar moiety position as protopanaxadiols (Rb1, Rc, Rd) and protopanaxatriols (Rg1, Re). In T2DM models, ginsenosides enhance insulin secretion, improve peripheral insulin sensitivity via PI3K/Akt/GLUT4 upregulation, attenuate oxidative stress and advanced glycation end-product formation, and modulate gut microbiota composition [53]. Ginsenoside Rb1 specifically activates AMPK in skeletal muscle, while Rg1 protects pancreatic β-cells from glucotoxic apoptosis by upregulating the Bcl-2/Bax ratio [53].

#### 4.3.3. Fenugreek-Derived Compounds (*Trigonella foenum-graecum*)

The antidiabetic activity of fenugreek seeds derives from multiple distinct phytochemical fractions: (i) galactomannan (a heteropolysaccharide comprising a β-1,4-mannose backbone with α-1,6-galactose branches), which delays gastric emptying, reduces the glycaemic index of co-ingested carbohydrates, and enhances GLP-1/GIP secretion; (ii) steroidal saponins principally diosgenin and its furostanol glycosides, which inhibit α-glucosidase and enhance GLUT4 expression; and (iii) trigonelline (N-methylnicotinic acid; a pyridinium alkaloid), which inhibits gluconeogenic enzymes and restores pancreatic β-cell function in STZ-treated rodents [54]. Preclinical and clinical studies confirm that fenugreek seed supplementation reduces fasting and postprandial blood glucose, improves insulin sensitivity, and lowers HbA1c with good tolerability [54].

#### 4.3.4. *Momordica charantia* (Bitter Melon) Bioactives

The hypoglycaemic activity of *Momordica charantia* is attributed to three distinct bioactive classes: charantin (a steroidal saponin mixture of β-sitosterol glucoside and stigmasterol glucoside, which activates GLUT4 and AMPK in skeletal muscle), polypeptide-p (an insulin-mimetic plant polypeptide that activates insulin receptors in peripheral tissues), and momordicin/momordicilin-type cucurbitane triterpenoids, which inhibit α-glucosidase and gluconeogenic phosphatases [55]. Whole-fruit preparations, juice, and standardised extracts have demonstrated reductions in fasting glucose and improvement in glucose tolerance in clinical studies in South and Southeast Asian populations, though evidence for superiority over metformin in randomised trials is limited [55].

#### 4.3.5. Cinnamon-Derived Phytochemicals (*Cinnamomum* spp.)

Cinnamon bark contains two major antidiabetic phytochemical classes: cinnamaldehyde ((E)-3-phenylprop-2-enal; a phenylpropanoid), which stimulates pancreatic insulin secretion via insulinotropic effect, activates PPAR-γ (peroxisome proliferator-activated receptor gamma) and AMPK in preadipocytes, suppresses pro-inflammatory cytokines, and reduces lipid peroxidation in islet cells; and procyanidins (B-type proanthocyanidins, condensed tannins), which enhance insulin receptor β-subunit autophosphorylation, inhibit protein tyrosine phosphatase 1B (PTP1B), and improve post-receptor insulin signal transduction [41]. Meta-analyses of randomised controlled trials show modest but significant reductions in fasting blood glucose (~8–10 mg/dL) and improvements in lipid profiles with cinnamon supplementation [41,56].

**Table 1:** Key antidiabetic phytochemicals: plant source, bioactive class, molecular targets, and preclinical/clinical evidence summary.

Phytochemical	Chemical Structure	Plant Source	Corrected Chemical Class	Key Antidiabetic Mechanisms	Preclinical Evidence Summary	Clinical Evidence Summary	Ref.
Curcumin	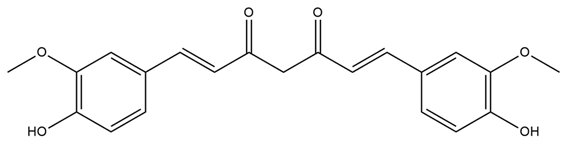	*Curcuma longa* L. (rhizome)	Curcuminoid (diarylheptanoid); principal curcuminoid; β-diketone tautomer (enol form predominates in solution)	PI3K/Akt/GLUT4 ↑; NF-κB/IKKβ/JNK ↓; SIRT1 ↑; α-glucosidase ↓; β-cell regeneration; Nrf2 ↑	In HepG2 and 3T3-L1 cells: GLUT4 translocation ↑, gluconeogenic gene expression ↓; in STZ-induced diabetic rats: FBG ↓ ~40–55%, β-cell mass preservation ↑, inflammatory markers ↓ [48,57]	Meta-analysis of 59 RCTs: FBS ↓ 4.60 mg/dL, HbA1c ↓ 0.32%, HOMA-IR ↓ 0.33; complementary therapy for disturbed glycaemia [51]	[33,47,48,57,58]
Berberine	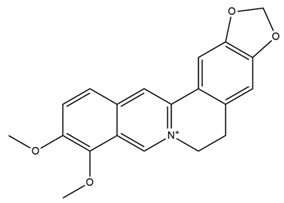	*Berberis aristata* DC., *Coptis chinensis* Franch., *Phellodendron amurense* Rupr.	Isoquinoline alkaloid-protoberberine type; quaternary ammonium salt	AMPK ↑ (complex I ↓); hepatic gluconeogenesis ↓ (PEPCK/G6Pase ↓); GLUT1/4 ↑; NF-κB ↓; IRS-1 tyrosine phosphorylation ↑; insulin sensitisation	In db/db mice and HFD rodent models: FBG ↓ ~35–45%, insulin sensitisation comparable to metformin; hepatic lipid accumulation ↓; AMPK activation confirmed in multiple tissue models [27,34]	Umbrella meta-analysis of RCTs: FBG ↓ (ES −0.65 to −0.77), HbA1c ↓ (ES −0.57), HOMA-IR ↓ (ES −0.71–1.04), IL-6 ↓, TNF-α ↓, CRP ↓; efficacy comparable to metformin in some trials [59]	[27,32,34,59]
Quercetin	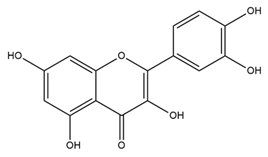	*Allium cepa* L., *Camellia sinensis* (L.) Kuntze, *Fagopyrum esculentum* Moench	Flavonoid-flavonol subclass; 3,3′,4′,5,7-pentahydroxyflavone	α-glucosidase ↓; DPP-4 ↓; GLP-1 ↑; PI3K/Akt/GLUT4 ↑; SIRT1 ↑; β-cell protection	In STZ-induced rats: FBG ↓, DPP-4 activity ↓, GLP-1 ↑, NLRP3 inflammasome ↓, antioxidant enzymes ↑ [36]; in Caco-2 cells: glucose uptake ↓ via SGLT1 inhibition [29]	Umbrella review (18 RCTs): insulin level ↓ (WMD −1.07 μIU/mL); SBP ↓; fasting plasma glucose not significantly changed; more long-duration trials needed [59]	[6,29,36,47,59]
Resveratrol	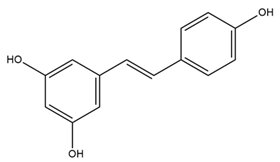	*Vitis vinifera* L., *Polygonum cuspidatum* Sieb. and Zucc.	Stilbenoid-trans-stilbene; (E)-3,5,4′-trihydroxystilbene	SIRT1/AMPK/FOXO1 axis; GLUT4 ↑; hepatic gluconeogenesis ↓; ROS ↓; SIRT1-deacetylation of PGC-1α (peroxisome proliferator-activated receptor gamma coactivator-1 alpha)	In insulin-resistant 3T3-L1 adipocytes: SIRT1 ↑, p-AMPK ↑, GLUT4 membrane accumulation ↑, resistin ↓; in HFD/STZ mice (CS/Res/SeNP): FBG ↓, IR ↓, dyslipidaemia ↓, anti-apoptotic effects via PI3K/Akt/mTOR [44,46]	Clinical evidence is inconsistent; modest reductions in fasting glucose and HbA1c in some small trials; mechanistic cardioprotective effects in T2DM-associated CV complications reviewed [38]; no registered Phase II/III RCT data for nanoformulated resveratrol	[44,45,46,57]
EGCG (Epigallocatechin-3-gallate)	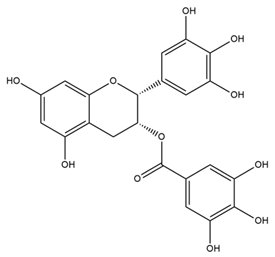	*Camellia sinensis* (L.) Kuntze (green tea leaf)	Flavonoid-flavan-3-ol subclass (catechin); (−)-epigallocatechin-3-O-gallate	α-glucosidase ↓; DPP-4 ↓; SGLT1/2 ↓; AMPK ↑; gut microbiota (*Akkermansia muciniphila*, *Lactobacillus* spp.) ↑; intestinal permeability ↓; β-cell protection	Addresses 8 of the “ominous octet” pathophysiological defects of T2DM in in vitro/rodent models; GLUT4 ↑ in skeletal muscle; intestinal inflammation ↓; renal glucose reabsorption ↓ [48]	Emerging clinical evidence for glycaemic control; high inter-individual variability due to poor bioavailability; dose optimisation and nanoformulation needed before conclusive clinical trials [48]	[48,60]
Gymnemic Acids	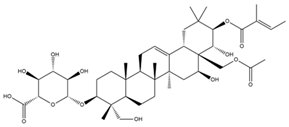	*Gymnema sylvestre* R.Br. (leaves)	Oleanane-type triterpenoid saponin (oleanolic acid glycosides; C30 triterpenoid backbone with 1–3 sugar units)	Intestinal glucose absorption ↓ (brush-border binding); sweet-taste receptor ↓; α-glucosidase ↓; GLP-1 ↑; β-cell regeneration ↑; pancreatic insulin output ↑	In STZ-induced rats and diabetic mouse models: FBG ↓, islet morphology improved, β-cell mass ↑; gymnemic acid IV found most potent for α-glucosidase inhibition; *G. sylvestre* extract protects β-cells from alloxan-induced apoptosis [47,51]	Systematic review of clinical studies: FBG ↓, postprandial glucose ↓, HbA1c ↓ in T2DM patients; body weight reduction in some trials; effect size smaller than in preclinical data [51]	[35,47,51]
Andrographolide	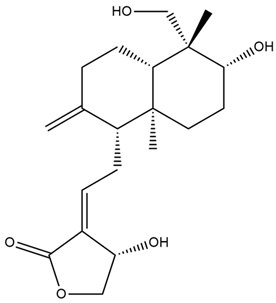	*Andrographis paniculata* (Burm.f.) Nees (aerial parts)	Bicyclic diterpenoid lactone-labdane-type diterpene; α-methylene-γ-butyrolactone moiety (Michael acceptor)	NF-κB covalent inhibition (p50 Cys38 alkylation); PI3K/Akt/GSK-3β ↑; β-cell protection; α-glucosidase ↓; PEPCK ↓	In STZ-induced T1DM and T2DM rodents: FBG ↓ significantly, pancreatic islet morphology preserved, oxidative stress ↓, insulin output ↑; andrographolide (10 mg/kg): FBG ↓ 45–52% vs. diabetic controls [50,51]	No registered Phase I/II clinical trial for nanoformulated andrographolide; isolated Phase I safety data for crude extract exist in healthy volunteers; adequate human T2DM efficacy data not yet available	[49,50,51]
Ginsenosides	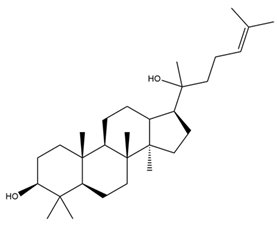	*Panax ginseng* C.A. Mey., *Panax notoginseng* (Burkill) F.H.Chen (root)	Dammarane-type tetracyclic triterpenoid saponin-protopanaxadiol series (Rb1, Rc, Rd) and protopanaxatriol series (Rg1, Re)	PI3K/Akt/GLUT4 ↑; AMPK ↑ (Rb1 in skeletal muscle); β-cell protection (Rg1, Bcl-2/Bax ↑); oxidative stress ↓; gut microbiota modulation; GLP-1R activation	Rb1 activates hepatic AMPK, reduces gluconeogenesis; Rg1 protects β-cells from glucotoxic apoptosis; compound K (metabolite) stimulates insulin secretion in INS-1 cells; multiple rodent models confirm FBG ↓ and insulin sensitivity ↑ [53]	Several small clinical trials: ginsenoside Rg3 and compound K formulations improve insulin sensitivity and FBG in T2DM patients; overall evidence limited by small sample sizes and heterogeneous preparations [53]	[27,53]
Galactomannan	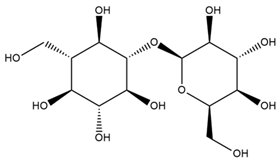	*Trigonella foenum-graecum* L. (seeds)	Galactomannan heteropolysaccharide (β-1,4-D-mannose backbone; α-1,6-D-galactose branches; M:G ratio ~1:1)	Viscosity ↑ → gastric emptying ↓; GLP-1/GIP ↑; α-amylase/maltase ↓; glucose diffusion rate ↓; gut microbiota modulation	In STZ-induced diabetic rats: postprandial glucose ↓, GLUT4 ↑, hexokinase ↑, G-6-Pase ↓; β-cell protection confirmed histologically [53]	Multiple RCTs: FBG ↓, 2 h-PPG ↓, HbA1c ↓; improvements in insulin resistance and lipid profiles; fenugreek seed (25–100 g/day) or extract well-tolerated [54]	[53]
Diosgenin/Steroidal Saponins	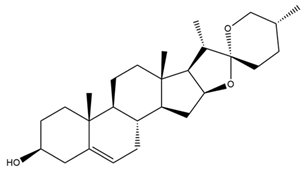	*Trigonella foenum-graecum* L. (seeds)	Steroidal saponin-furostane/spirostane type (C27 steroid nucleus; sugar moiety at C3 or C26)	α-glucosidase ↓; GLUT4 ↑; insulin sensitisation; cholesterol synthesis ↓	Steroidal saponins isolated from fenugreek: α-glucosidase IC_50_ values in the μg/mL range; diosgenin reduces hepatic cholesterol and improves glucose tolerance in HFD rodents [53]	Included in clinical outcomes of fenugreek seed supplementation trials; isolated steroidal saponin Phase II data lacking [53]	[53]
Trigonelline	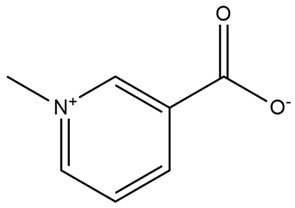	*Trigonella foenum-graecum* L. (seeds)	Pyridinium alkaloid-betaine derivative (N-methylnicotinic acid; zwitterionic)	Gluconeogenic enzyme ↓ (PEPCK, G6Pase); β-cell restoration; neuroprotection (nerve growth factor ↑); DPP-4 ↓	In STZ-induced diabetic rats: FBG ↓, insulin ↑, pancreatic histology improved; PEPCK mRNA ↓; sciatic nerve function improvement reported	Limited isolated clinical data; effects observed in fenugreek seed trials partly attributed to trigonelline; dedicated RCTs of isolated trigonelline in T2DM absent [48]	[53]
Charantin	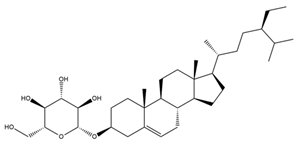	*Momordica charantia* L. (fruit)	Steroidal saponin mixture (β-sitosterol-3-O-β-D-glucoside + stigmasterol-3-O-β-D-glucoside; ~1:1)	GLUT4 ↑ (skeletal muscle and adipose); AMPK ↑; insulin receptor signalling ↑; gluconeogenesis ↓	In STZ rats: blood glucose ↓ ~30–50%, hepatic glycogen ↑, muscle glucose uptake ↑; charantin isolated fraction superior to whole juice in some models [54]	Whole-fruit preparations and extracts: modest reductions in FBG and PPG in T2DM patients in South/Southeast Asian trials; inferiority vs. metformin in head-to-head comparison [54]	[54]
Polypeptide-p	-	*Momordica charantia* L. (seeds)	Insulin-mimetic plant polypeptide (35 amino acid residues; structural homology to insulin B-chain)	Insulin receptor binding ↑; peripheral glucose uptake ↑; hepatic glycogenesis ↑; independent of pancreatic β-cells	In insulin-deficient rodent models: glucose lowering comparable to exogenous insulin at ~50 μg/kg s.c.; glycogen synthesis ↑ in liver and muscle [54]	Subcutaneous injections reported to lower blood glucose in T2DM patients in early Indian studies; oral bioavailability negligible due to proteolytic degradation; no recent Phase II RCT [54]	[54]
Cinnamaldehyde	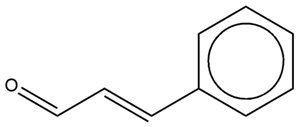	*Cinnamomum cassia* (L.) J.Presl, *C. verum* J.Presl (bark)	Phenylpropanoid-(E)-3-phenylprop-2-enal; α,β-unsaturated aldehyde derived from cinnamic acid	Pancreatic insulin secretion ↑ (insulinotropic); PPAR-γ ↑; AMPK ↑; pro-inflammatory cytokine ↓; lipid peroxidation ↓; islet antioxidant enzymes ↑	In STZ/alloxan-induced rodents: FBG ↓ ~25–40%; in vitro: adipocyte differentiation ↓, lipid accumulation ↓, NO production ↑ via eNOS; multiple METS pathways inhibited [55]	Systematic review: anti-METS activity (antidiabetic, antidyslipidaemic, anti-obesity) confirmed preclinically; robust RCT data in humans absent; dose, pharmacokinetics, and safety in humans require clinical validation [50]	[55]
Procyanidins (Cinnamon)	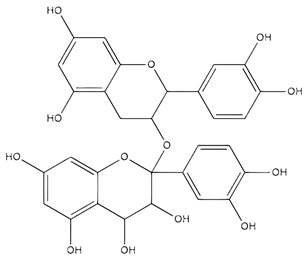	*Cinnamomum cassia* (L.) J. Presl (bark)	Proanthocyanidin-B-type condensed tannin (flavan-3-ol oligomers, mainly catechin/epicatechin dimers linked C4 → C8 or C4 → C6)	Insulin receptor β-subunit autophosphorylation ↑; PTP1B ↓ (post-receptor insulin signal ↑); glucose transport ↑ in muscle cells	In 3T3-L1 adipocytes and L6 muscle cells: insulin-mimetic effect; PTP1B inhibition IC_50_~50 μg/mL; rodent HFD models: glucose tolerance ↑, dyslipidaemia ↓ [55]	Cinnamon extract (procyanidin-enriched): meta-analyses show FBG ↓ ~8–10 mg/dL in T2DM RCTs; HbA1c effects inconsistent across trials; standardisation of procyanidin content in commercial preparations required [50]	[55]

Abbreviations: ↑, increase/upregulation; ↓, decrease/downregulation; AMPK, AMP-activated protein kinase; DPP-4, dipeptidyl peptidase-4; ER, endoplasmic reticulum; FBG, fasting blood glucose; G6Pase, glucose-6-phosphatase; GLP-1R, glucagon-like peptide-1 receptor; GLUT4, glucose transporter type 4; GSK-3β, glycogen synthase kinase-3β; HbA1c, glycated haemoglobin; HFD, high-fat diet; IKKβ, inhibitor of κB kinase β; IRS-1, insulin receptor substrate-1; JNK, c-Jun N-terminal kinase; MHCP, methylhydroxychalcone polymer; NF-κB, nuclear factor kappa-B; Nrf2, nuclear factor erythroid 2-related factor 2; PEPCK, phosphoenolpyruvate carboxykinase; PPAR-γ, peroxisome proliferator-activated receptor gamma; PPG, postprandial glucose; RCT, randomised controlled trial; SGLT, sodium-glucose co-transporter; SIRT1, sirtuin-1; STZ, streptozotocin; TG, triglycerides; TNF-α, tumour necrosis factor-alpha.

## 5. Biopharmaceutical Barriers to Phytochemical Delivery and the Nanoformulation Imperative

**Figure 3:**
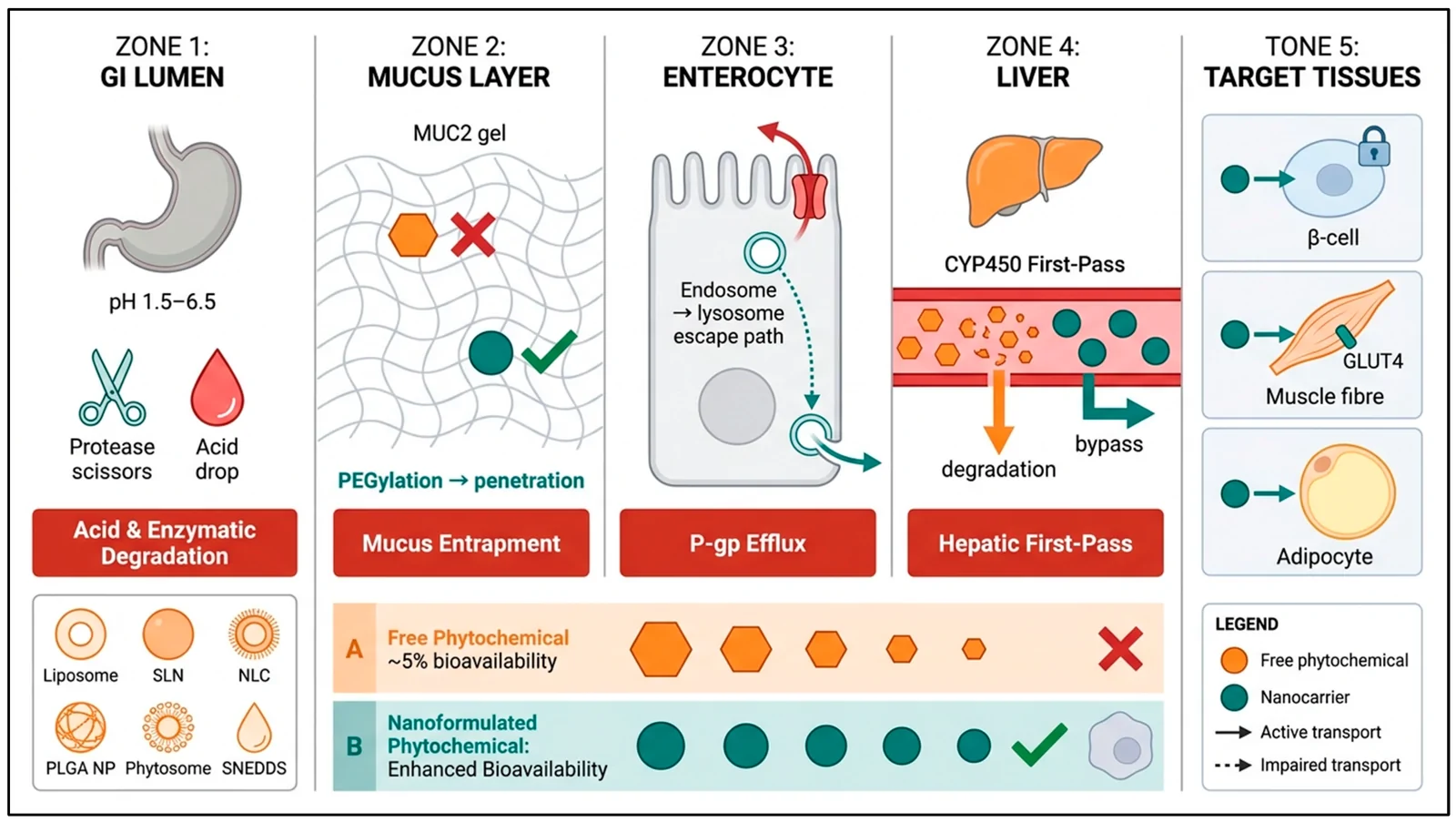
Biopharmaceutical barriers and nanocarrier solutions along the oral absorption pathway.

All oral bioavailability enhancement ratios cited in Section 5 and Section 6 are derived from preclinical pharmacokinetic studies in rodent models unless explicitly labelled [CN]. No head-to-head human pharmacokinetic comparison of nanoformulated versus conventional phytochemicals in T2DM has been registered or published at the time of this review. The pharmacological richness documented for antidiabetic phytochemicals in the preceding section contrasts sharply with the clinical outcomes reliably achieved by conventional oral formulations of these compounds. This disparity is not primarily a reflection of inadequate potency at molecular targets; rather, it reflects the imposition of a series of sequential biopharmaceutical barriers that substantially attenuate the fraction of the administered dose reaching systemic circulation in its pharmacologically active form [8,61,62]. Understanding these barriers and the structural and physicochemical properties that render individual phytochemicals susceptible to them is a prerequisite for both the rational design of nanocarrier delivery systems and anticipating which compound–carrier pairings are most likely to yield clinically meaningful improvements in therapeutic outcomes [42,61,62,63] (Figure 3).

### 5.1. Solubility, Stability, and First-Pass Metabolism Challenges: A Pharmacokinetic Perspective

These oral delivery properties follow the general pharmacokinetic principles for any drug administered by mouth, but most antidiabetic phytochemicals fail to meet multiple requirements for desirable oral delivery, which makes this a highly challenging class of drug candidates. Poor aqueous solubility is typically the first obstacle encountered. Of these, curcumin is best characterised and has an aqueous solubility of ~11 ng/mL at physiological pH, much lower than needed for dissolution-limited absorption to achieve therapeutic levels after standard oral doses. This solubility consideration is due to the hydrophobic nature of the long conjugated λ-electrons and is exacerbated by its pH-dependent instability whereby the β-diketone structure in the molecule is rapidly broken down in the alkaline intestinal pH levels above 7.0, with the breakdown products being mainly ferulic acid and feruloyl methane, the latter of which does not have the same antidiabetic activity as curcumin. Resveratrol is somewhat less soluble but turns into the less active cis conformation after a quick photoisomerisation process at physiological light and pH values. EGCG is also labile in its other reported properties, such that ring-opening and epimerisation within 80% of its structure occur in 30 min under pH 7.4 and 37 °C conditions, which closely model the small intestinal lumen in the human body [9,62,63]. After blood flow through the stomach and intestine, gastric and intestinal epithelial absorption is a second round of challenges when a phytochemical is not chemically modified and reaches the intestine in a soluble form. The first line of defence against the gastrointestinal mucosa is its mucus gel layer, a dense viscoelastic network comprising MUC2 mucin-2 glycoprotein that is secreted by goblet cells, and this layer lies above the absorptive epithelium. Poorly wettable compounds are hydrophobic, tend to aggregate and get trapped in this gel, which decreases the effective diffusion of hydrophobic compounds to the brush border. The membrane that faces the enterocyte’s apical membrane must sufficiently facilitate membrane permeability for phytochemicals to passively diffuse across the membrane for transcellular absorption, and if the phytochemical does reach the apical membrane, it must possess the proper characteristics in terms of lipophilicity and size. Multiple hydroxyl substituents will reduce partitioning across the membrane, which also hinders passive diffusion of polyphenols.

Claudin and occludin tight junctions provide a molecular mass cutoff of ~200–500 Da, and under normal physiological conditions, most phytochemicals having molecular weights above this cutoff are restricted from paracellular transport. Efflux due to P-glycoprotein (also known as ABCB1), an ATP-dependent transporter that spans the cell membrane and is strongly expressed on the apical surface of the intestinal enterocyte, is also an epithelial-level barrier and is perhaps the most important for several important phytochemicals. If the cell’s active pumping capacity is adequate, the net transcellular flux from the enterocytes towards the gut lumen (trough) will be a small fraction of the apparent apico-lateral concentration gradient (trough) because the cell’s active pumping capacity drives the substrate from the cell into the enzyme environment of the intestinal lumen. As mentioned above, in humans there is a thermodynamic barrier, a solubility barrier, and the fact that both berberine and some of its derivatives are high-affinity P-gp (P-glycoprotein) substrates, which also results in a large efflux due to MRP2 (multidrug resistance-associated protein 2) [64,65]. The intestinal epithelium is the primary barrier that phytochemicals must pass to reach portal circulation and the liver, which has the hepatic first-pass metabolising system, the most clinically important single barrier for phytochemicals. The cytochrome P450 enzyme family, especially CYP3A4 (cytochrome P450 3A4) and CYP1A2 (cytochrome P450 3A4), catalyses oxidative transformations of polyphenols, terpenoids and alkaloids to form phase I metabolites that are generally not as lipophilic and, in most cases, less pharmacologically active than the parent compound. There are three types of phase II conjugations: glucuronidation (UGT enzymes), sulfation (SULT enzymes), and methylation (COMT enzymes). The products (hydrophilic conjugates) are excreted quickly in urine/bile. Reduction in phase I, for instance, converts curcumin to hexahydrocurcumin and then glucuronidation and sulfation ensue in phase II conjugation; free curcumin is found at practically undetectable amounts in plasma after oral administration to humans of conventional formulations, with the conjugated species contributing to more than 90% of the detectable plasma species. Quercetin is conjugated in a similar way to flavonols, mainly to the C3 position as a glucuronide and resulting in less than 20% of an oral dose appearing in systemic circulation as the aglycone.

An additional pharmacokinetic–pharmacodynamic complexity with berberine is the fact that the parent compound is metabolised to berberrubine (apo-berberine) and other lipophilic metabolites by intestinal first-pass metabolism via CYP3A4, which have a significantly different organic function profile than the parent compound [64,65]. Most of these antidiabetic phytochemicals have pharmacokinetics that are such a limiting factor that the consistent formulation of a reproducible tissue level with a therapeutic effect is virtually impossible without intervention, as they have only 0.3–5% bioavailability in conventional oral formulations. The combined effect of these drawbacks is a pharmacokinetic profile that is low in the case of most antidiabetic phytochemicals, with short t½, high inter-individual variability of plasma concentrations and reduced plasma AUCs (area under the plasma concentration–time curve) (compared to the plasma exposure required to produce the documented plasma antidiabetic effects in preclinical systems). This is not just hypothetical since there is an inherent gap between promising in vitro and rodent-based data, and the mixed, and frequently negative, results seen in human clinical trials for such phytochemicals as curcumin and resveratrol. There are several complementary mechanisms which mitigate the deleterious effects of chemical degradation of labile molecules during delivery, which are provided by nanocarrier-based delivery systems: delivery of lipophilic molecules into lipid or polymer matrices, endocytic mechanisms of uptake without recognition by P-gp, and a decrease in renal clearance of encapsulated molecules, thereby resulting in an increase in half-life for systemic circulation. The fact that available evidence suggests that bioavailability of phytochemicals loaded into an appropriately designed nanocarrier can be 5- to 10-fold higher than the bioavailability of the native phytochemical is enough to take cancer efficacy from the preclinical to human pharmacokinetic profile [8,13,61,66].

### 5.2. Structure–Activity Relationships Governing Bioavailability: A Molecular Descriptor Framework

The barriers to oral absorption outlined in Section 4.1 do not affect all antidiabetic phytochemicals equally; the relative magnitude of solubility limitation, membrane permeability restriction, transporter-mediated efflux, and metabolic vulnerability is instead dictated by a small set of structural and physicochemical descriptors intrinsic to each compound class. Framing bioavailability in terms of these descriptors, rather than restating the sequential barrier walkthrough, allows the dominant liability of a given phytochemical to be anticipated from its molecular architecture and provides the rational basis on which specific nanocarrier chemistries are subsequently matched to specific compounds in Section 5 [8,61,64,67]. Molecular weight and the paracellular/passive-diffusion threshold. Antidiabetic phytochemicals span an unusually wide molecular weight range for a single pharmacological class. Small phenolic monomers such as curcumin (MW ≈ 368 Da) and the quercetin aglycone (MW ≈ 302 Da) fall within the size range compatible with transcellular passive diffusion, provided lipophilicity is adequate [9,10,11,13]. By contrast, the intact triterpenoid glycosides that constitute gymnemic acid preparations and the dammarane-type ginsenosides (MW commonly in the 800–950 Da range as parent glycosides) and the high-molecular-weight galactomannan fraction of fenugreek lie well outside both the paracellular tight-junction size cutoff and the practical ceiling for efficient passive transcellular diffusion [49,52,53]. For this glycosylated group, oral bioavailability is not primarily a solubility or first-pass metabolism problem in the sense described for curcumin or resveratrol, but a size-exclusion problem: mechanistic activity in vivo depends substantially on colonic microbial deglycosylation of the parent saponin to smaller, absorbable aglycones and secondary metabolites, most notably the conversion of protopanaxadiol ginsenosides to Compound K by gut microbiota, rather than on direct absorption of the administered glycoside [52]. This is a structurally distinct bioavailability mechanism from the pH-instability and CYP-mediated metabolism problems that dominate for the small phenolic monomers, and it carries direct formulation implications: nanocarrier strategies for saponin-rich extracts must contend with microbiome-dependent bioconversion kinetics rather than simple protection from gastric or hepatic degradation [52,53].

Lipophilicity and hydrogen-bond donor count: Among the small-molecule phytochemicals, logP and hydrogen-bond donor (HBD) count differentiate membrane permeability independently of aqueous solubility. Curcumin’s moderate lipophilicity is sufficient for passive membrane partitioning once solubilised, such that its principal bioavailability constraint is dissolution rate and first-pass conjugation rather than membrane permeability itself [9,11,13]. Quercetin and EGCG, by contrast, carry considerably more hydroxyl substituents on their flavonoid and catechin-gallate scaffolds, respectively; this higher polar surface area and HBD count place both compounds in a regime of comparatively restricted passive transcellular diffusion even where local solubility is adequate, a structural constraint that compounds their already well-documented susceptibility to conjugation and oxidative degradation [46,49,63]. Resveratrol’s stilbene scaffold, with only three hydroxyl groups arranged on a rigid diphenylethylene core, occupies an intermediate position, and its principal SAR-linked vulnerability is not permeability but the photoisomerizable central double bond, which converts the active trans isomer to the pharmacologically inactive cis form under ordinary light exposure [44,45,57].

Charge state and transporter recognition: Berberine’s permanently ionised quaternary ammonium isoquinoline structure represents a distinct SAR category: cationic charge at physiological pH confers high-affinity recognition by P-glycoprotein and related efflux transporters, which is the principal determinant of its exceptionally low reported oral bioavailability (0.36–0.68%), rather than aqueous solubility, which is comparatively less limiting for this alkaloid than for curcumin or EGCG [34,64,65]. Andrographolide’s α-methylene-γ-butyrolactone moiety confers a different SAR-defined liability: its electrophilic lactone ring, while central to its covalent NF-κB inhibitory mechanism, is also the site of rapid hepatic metabolic attack, linking pharmacological mechanism and metabolic vulnerability to the same structural feature [50,51].

Implications for carrier matching: This descriptor-based classification indicates that no single nanocarrier rationale applies uniformly across the antidiabetic phytochemical class. Compounds whose limitation is predominantly one of dissolution and lipophilic partitioning (curcumin, resveratrol) are well matched to lipid-based carriers that solubilise the payload within a lipophilic matrix [9,10,11,13,45,57]. Compounds whose limitation is P-gp-mediated efflux recognition (berberine) benefit disproportionately from carriers that enable endocytic cellular uptake by passing apical transporter recognition altogether, independent of any improvement in intrinsic solubility [34,65,67]. Compounds whose limitation is a combination of high polarity and rapid conjugation (quercetin, EGCG) require carriers that simultaneously improve membrane contact time (mucoadhesion) and shield the molecule from luminal and hepatic conjugating enzymes [46,49,63]. Compounds whose limitation is fundamentally one of molecular size and glycosylation state (gymnemic acids, ginsenosides, fenugreek galactomannans) are less amenable to conventional solubility- or efflux-directed nanocarrier strategies and instead require delivery systems engineered around microbiome-targeted or colon-specific release kinetics [50,52,53]. This structure-to-carrier logic, rather than a restatement of the pharmacokinetic barriers themselves, is what determines the nanoformulation strategies reviewed in Section 5 [8,13,61].

## 6. Advanced Drug Delivery Systems for Phytochemical Optimisation in Type 2 Diabetes Mellitus

Advanced drug delivery systems for phytochemical optimisation in T2DM represent one of the most pressing public health challenges of the twenty-first century, affecting over 500 million individuals worldwide and placing an immense burden on healthcare systems globally. While conventional pharmacological interventions remain the cornerstone of T2DM management, growing evidence has highlighted the therapeutic potential of plant-derived bioactive compounds, collectively referred to as phytochemicals, in modulating key pathological mechanisms underlying the disease. Compounds such as curcumin, berberine, quercetin, resveratrol, and epigallocatechin gallate (EGCG) have demonstrated promising antidiabetic properties in preclinical and clinical investigations, acting through diverse mechanisms including the enhancement of insulin sensitivity, inhibition of alpha-glucosidase activity, attenuation of oxidative stress, and suppression of chronic low-grade inflammation [5,6,12]. Despite their considerable therapeutic promise, the clinical translation of phytochemicals into effective antidiabetic agents has been substantially impeded by several inherent pharmacokinetic limitations. Chief among these are poor aqueous solubility, rapid gastrointestinal degradation, extensive first-pass hepatic metabolism, and low oral bioavailability, which collectively limit the ability of these compounds to achieve and sustain therapeutically meaningful plasma concentrations. For instance, curcumin, perhaps the most extensively studied phytochemical in metabolic disease research, exhibits less than 1% oral bioavailability in its native form, rendering conventional formulation approaches largely inadequate for clinical use [9,13].

In response to these challenges, the field of pharmaceutical nanotechnology has emerged as a transformative platform for the rational reformulation of phytochemicals. Advanced drug delivery systems are designed to encapsulate, protect, and transport bioactive molecules in a manner that overcomes physicochemical barriers, prolongs systemic circulation, enables controlled or targeted release, and ultimately improves therapeutic efficacy at reduced doses. Over the past two decades, a broad spectrum of nanocarrier architectures has been developed and evaluated for phytochemical delivery, spanning lipid-based systems, polymeric nanoparticles, and colloidal dispersions [11,12,61]. These platforms differ substantially in their physicochemical properties, loading capacities, release kinetics, and mechanisms of cellular interaction, yet they share a common objective: maximising the biopharmaceutical performance of otherwise poorly bioavailable phytochemicals. This section provides a comprehensive and critical review of the principal advanced drug delivery systems that have been investigated for the optimisation of phytochemical delivery in the context of T2DM. The discussion is structured to address three major categories of delivery platforms. The first encompasses lipid-based carriers, specifically liposomes, SLNs, and NLCs, which leverage the amphiphilic nature of lipid molecules to encapsulate both hydrophilic and lipophilic phytochemicals within well-defined particulate architectures [11,61]. The second category examines polymeric nanoparticles, including systems based on PLGA, chitosan, and polyethylene glycol (PEG)-functionalised polymers, as well as hybrid polymer–lipid nanoplatforms that combine the structural advantages of both material classes [66,68]. The third category covers colloidal and self-assembling systems, namely nanoemulsions, SNEDDS, and phytosomes, each of which offers distinct formulation strategies to enhance solubilisation, membrane permeation, and gastrointestinal absorption [9,61].

By examining the design principles, formulation strategies, mechanistic advantages, and experimental outcomes associated with each delivery platform, this section aims to provide a coherent framework for understanding how nanotechnology-enabled approaches can bridge the gap between the demonstrated in vitro bioactivity of phytochemicals and their in vivo therapeutic realisation in T2DM. Such an understanding is essential not only for advancing the scientific basis of phytochemical-based antidiabetic therapy but also for guiding the rational development of next-generation nanomedicines in the management of metabolic disease [12,61] (Table 2).

### 6.1. Lipid-Based Carriers: Liposomes, Solid Lipid Nanoparticles (SLNs), and Nanostructured Lipid Carriers (NLCs)

The most widely investigated and clinically advanced class of poorly bioavailable bioactive compound drug delivery systems are lipid-based nanocarriers. They are readily adaptable, appreciable by biological systems and permit hydrophobic and hydrophilic payloads to be inserted into the construct, making them especially convenient for phytochemical delivery [12,61]. The physicochemical architectures of liposomes, solid lipid nanoparticles, and nanostructured lipid carriers each offer distinct advantages for T2DM therapy. Liposomes are self-assembled vesicles composed of one or more concentric phospholipid bilayers surrounding a hydrophilic core. This dual-compartment structure enables the simultaneous encapsulation of aqueous-soluble molecules within the aqueous interior and lipid-soluble molecules within the bilayer. Specifically, curcuminoids, quercetin and berberine have shown significantly better cellular uptake and enhanced plasma pharmacokinetics, as well as superior antihyperglycaemic effects when used in liposomal formulation as compared to their non-formulated counterparts in in vitro and in vivo studies [12,61].

Liposomes can fuse to the cell membrane and undergo endosomal internalisation, further enhancing delivery and accumulation within target tissues involved in T2DM pathophysiology, such as hepatocytes, skeletal muscle cells, and pancreatic beta cells. However, the existing liposomes have several disadvantages, such as physical instability, oxidizability of phospholipids and rather short circulation time in the bloodstream. Attempts have been made to resist some of these difficulties through modification of the surface by polyethylene glycol (PEGylation), providing steric stabilisation, and by the incorporation of cholesterol for its ability to enhance membrane rigidity [69,70]. The first generation of SLNs was developed in the early 1990s as a physical alternative to liposomes with better physical stability and scalability for industrial production [15,16]. The solid part of SLNs is normally made up of triglycerides, fatty acids, waxes or combinations of these and the matrix is stabilised by a compatible surfactant system. Since the lipid core is solid at physiological temperatures, drug mobility within the matrix is limited, yielding a sustained-release profile that helps maintain prolonged therapeutic concentrations of the phytochemicals. There are abundant studies that demonstrate how SLNs enhance the gastrointestinal stability and intestinal permeation of resveratrol, EGCG and piperine and maintain their antioxidant and anti-inflammatory activities in diabetic animal models [15,45]. It is, however, acknowledged that SLNs might undergo polymorphic changes during the storage process, resulting in the progressive leakage of encapsulated drug molecules over time and, consequently, a lower loading efficiency over time [15,61].

To overcome the crystallisation-related disadvantages of SLNs, such as crystallisation, a second-generation lipid nanoparticle system was developed, known as nanostructured lipid carriers (NLCs) [15,17]. NLCs consist of a mixture of solid and liquid lipids, which are formulated so they form a structurally imperfect, partially amorphous structure that will allow for a higher volume of accommodation for drug molecules [17,61]. This provides a better reduction in the order of a matrix, resulting in an increased loading capacity and superior long-term stability due to fewer polymorphic crystallisation events that lead to drug expulsion from SLNs [15,61]. The NLC formulations of curcumin-quercetin have shown excellent encapsulation efficiency, good mucoadhesive capacity and a better oral bioavailability profile in animal models of T2DM [17,55]. As a group, the lipid-based carrier family is a mature and well-validated carrier system for phytochemical optimisation; they have a regulatory history and are increasingly being demonstrated as useful in pathological studies related to metabolic disease applications [12,61].

### 6.2. Polymeric Nanoparticles (PLGA, Chitosan, PEGylated Systems) and Hybrid Polymer–Lipid Nanoplatforms

The polymeric nanoparticles are of great interest as a versatile carrier for phytochemical delivery because they possess structural tunability, capacity for surface functionalisation, and capability of imparting programmed pharmaceutical release. The properties of the polymers, such as type, molecular weight, degree of cross-linking, and surface chemistry, can be modified to achieve a variety of physicochemical and biopharmaceutical properties suitable for the delivery needs of each phytochemical agent, specifically for the treatment of T2DM [66,68]. The most widely used biodegradable polymer is poly(lactic-co-glycolic acid), which is registered and approved for a wide range of pharmaceutical nanotechnology applications. Under physiological conditions, PLGA nanoparticles hydrolyse to release the phytochemicals encapsulated within them in a sustained and predictable release based on the PLGA ratio and molecular weight. Such a controlled release profile is especially beneficial in helping to maintain the therapeutically relevant levels of less-soluble phytochemicals like curcumin, berberine, and quercetin at target sites known to play a role in T2DM (islets of Langerhans, liver, and insulin-sensitive peripheral tissues). Many studies have shown that PLGA encapsulation significantly improves the in vivo antidiabetic activity of these compounds, lowers the level of fasting blood glucose, increases insulin secretion and counteracts the increase in oxidative and inflammatory indicators in diabetic rodent models induced with streptozotocin [61,68].

Recently, the N-deacetylation of chitin to produce chitosan, a naturally occurring polysaccharide, has been identified as an especially promising polymeric drug delivery system for oral administration. On account of its cationic nature at physiological pH values found in the gastrointestinal tract, it allows electrostatic interaction with the glycoproteins in the digestive tract that have a negative charge at physiological pH levels, resulting in a high mucoadhesive effect in the gastrointestinal tract, which increases the in vivo residence time and the paracellular transport of drugs in the digestive tract. Chitosan nanoparticles have been shown to improve the relative (2 to 5-fold) bioavailability of quercetin, naringenin, and EGCG vs. free suspensions of phytochemicals when administered orally. Moreover, due to the large numbers of amino and hydroxyl groups on the chitosan backbone, which allow for easy chemical modification, and the enhancement of the biocompatibility and low toxicity of the polymer, targeted delivery strategies and stimuli-responsive delivery strategies can be developed. PEGylated polymeric nanoparticles carry the PEG molecules on their surfaces, which provide a steric repulsion barrier to decrease the rate of opsonisation and the extent of opsonisation, reduce recognition by the mononuclear phagocyte system, and significantly extend systemic circulation time. For the latter, the so-called stealth property is relevant when systemic bioavailability and tissue-level phytochemical uptake are “therapeutic goals” [55,65]. For instance, hybrids of polymer and lipid nanoplatforms have become an advanced and increasingly important strategy that aims at generating synergistic combinations of polymeric and lipid features in a single united carrier. A typical hybrid design makes use of a polymeric core material for its structural strength, controlled release properties, and high amount of drug loading, and a lipid shell/lipid coating for its biocompatibility, better affinity to membranes, and better internalisation. The use of hybrid nanoplatforms for the co-delivery of curcumin with other antidiabetic phytochemicals/traditional hypoglycaemic drugs has shown synergistic therapeutic effects and provides room for multi-modal T2DM management [61,71]. The concept of curcumin acting as a molecular synergy amplifier provides a compelling mechanistic rationale for the superior outcomes reported with hybrid co-delivery platforms [72,73]. This occurs through mechanistic complementarity with co-delivered bioactives, suppression of adaptive resistance networks, and pharmacokinetic synchronisation within nanocarrier systems. While this framework has been developed primarily in cancer research, its three core determinants multi-target engagement, resistance suppression, and synchronised exposure are directly applicable to the multi-pathway pathobiology of T2DM [73,74].

### 6.3. Nanoemulsions, Self-Nanoemulsifying Drug Delivery Systems (SNEDDS), and Phytosomes

**Figure 4:**
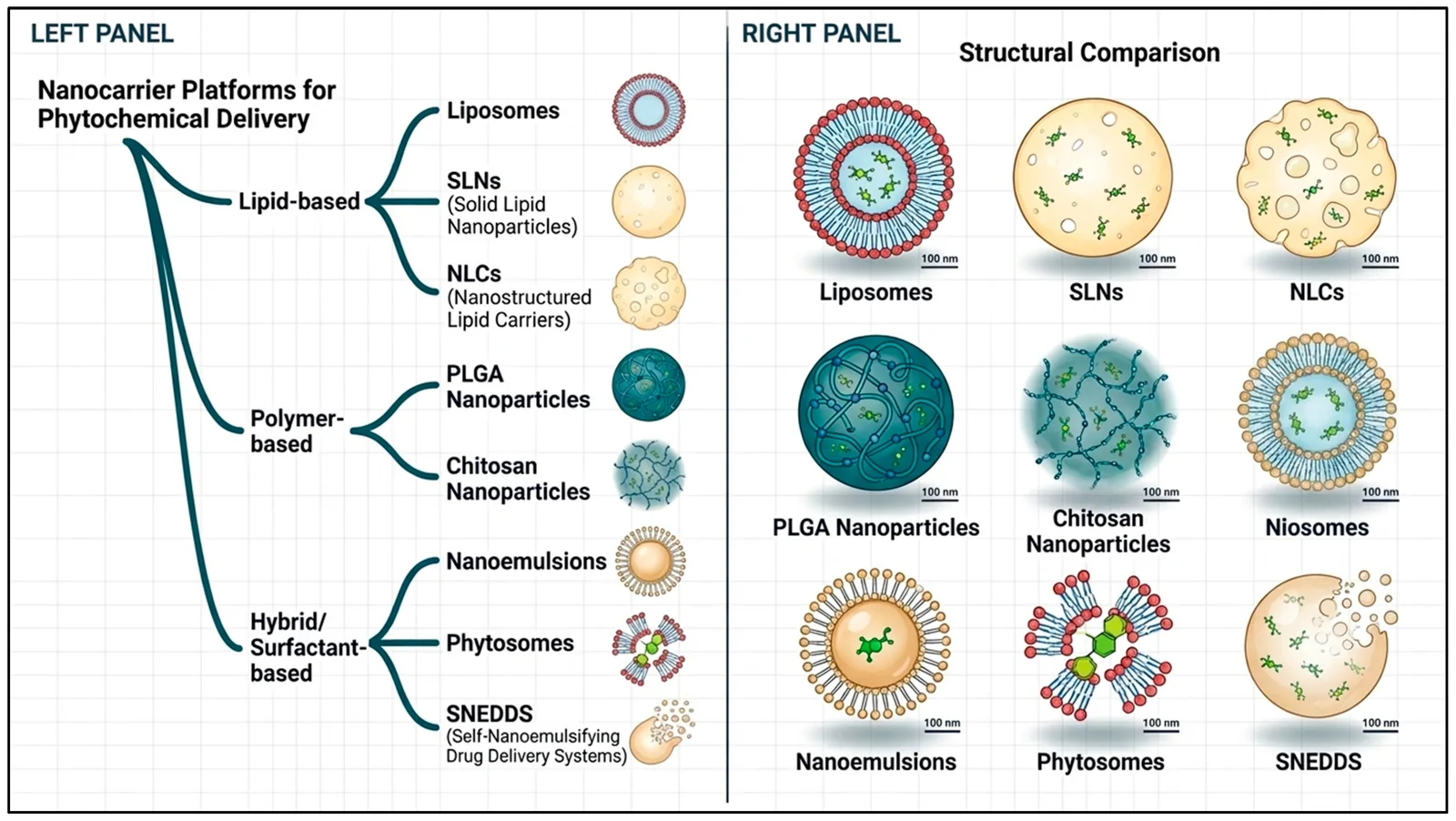
Classification tree and structural comparison of nanocarrier platforms for phytochemical delivery.

In addition to particulate nanocarriers, studies have examined delivery systems to overcome solubility and permeability limitations that limit the oral bioavailability of phytochemicals, such as colloidal and self-assembling systems. Three innovative technologies have been investigated FOR T2DM that are particularly effective for improving the bioavailability of phytolipophilic bioactive molecules: the biological approaches include nanoemulsions, SNEDDS, and phytosomes [72,73]. A nanoemulsion is an optically isotropic dispersion (thermodynamically or kinetically stable) of oil and water stabilised by an appropriate blend of surfactant and cosurfactant that typically has a droplet diameter from 20 to 200 nm. The very small droplet size provides a significantly increased interfacial surface area, allowing the capsule’s host lipophilic phytochemicals to be rapidly absorbed in the gastrointestinal tract and giving them a greatly enhanced apparent aqueous solubility [74]. Oil-in-water nanoemulsions of curcumin, resveratrol and thymoquinone have been prepared and shown to provide significantly improved dissolution rate and permeation across the intestine when compared to conventional oil dispersions [70,75,76]. Optimisation of nanoemulsion formulations using systematic experimental design approaches (Figure 4) is critical to determine the composition of the oil phase, the selection of a system for emulsifier, and the droplet size distribution of the formulation for good stability and biopharmaceutical performance. An interesting aspect of nanoemulsions is the self-nanoemulsifying drug delivery system (SNEDDS), which has an added advantage of spontaneous self-emulsification on contact with gastrointestinal fluid without a specific homogenisation or high-energy treatment process. SNEDDS consist of combinations of oils, non-ionic surfactants, cosurfactants, and the dissolved drug that self-emulsify to nanoscale oil-in-water upon gentle agitation in an aqueous phase (usually less than 200 nanometres) [72,73] (Table 2).

**Table 2:** Comparative overview of nanoformulation strategies for selected antidiabetic phytochemicals: encapsulation efficiency, particle size, release profile, and in vivo antidiabetic outcomes.

Nanocarrier Type	Phytochem. Payload	Preparation Method	Particle Size (nm)	Encapsulation Efficiency (%)	Drug Release Profile	In Vivo Antidiabetic Outcomes	Bioavailability Enhancement	Ref.
Solid Lipid Nanoparticle (SLN)	Curcumin	Solvent evaporation; stearic acid matrix; Brij 35 + Gelucire 48/16 (3:1) surfactant system, DoE-optimised	389.3 ± 9.95 nm	Not reported as a single value; loading efficiency enhanced by 3:1 Brij–Gelucire ratio	Sustained, Higuchi-model diffusion-controlled release; ~60% at 4 h, 80.1% at 6 h (vs. free CUR: ~60% at 30 min, 100% by end)	This study evaluated anticancer cytotoxicity in A549 lung adenocarcinoma cells (in vitro only). No diabetic animal model, no glycaemic endpoint, no antidiabetic claim can be attributed to this source	Not reported in vitro solubility enhancement only (452.5-fold increase in aqueous solubility, 0.25 → 111.2 µg/mL); no in vivo PK data	[15]
Chitosan-coated Solid Lipid Nanoparticle (SLN)	Berberine	Solvent-injection + high-pressure homogenisation; Witepsol 85E lipid core; chitosan (0.5 mg/mL) + Pluronic surfactant coating	295 ± 12 nm; Zeta +38 ± 1.1 mV; PDI 0.19 ± 0.09	88.2 ± 2.4% (lipid:drug ratio 1:0.9)	Sustained/biphasic release over 48 h: 82.9 ± 3.3% (SIF, pH 6.8), 46.1 ± 4.3% (SGF, pH 1.5); stable in SGF/SIF short-term with EE% decline to 63.9–72.7%	STZ-induced gestational diabetes mellitus (GDM) rat model (note: GDM, not standard T2DM). Dose-dependent ↓ FBG (203–267 mg/dL reduction), ↑ fasting insulin, improved HOMA-IR, OGTT (37–42% AUC ↓), ITT (52–66% AUC ↓), prevented body-weight loss, ↑ hepatic antioxidants (GSH/SOD/CAT/GPx), ↓ pro-oxidants (NO/MDA/MPO)-all significantly greater than free berberine	2.8-fold increase in relative oral bioavailability (AUC) vs. free berberine (healthy rats, 50 mg/kg). Cmax 399 ± 26 vs. 141 ± 35 ng/mL; Tmax 2 h vs. 0.5 h; AUC 2926 ± 75 vs. 692 ± 74 ng·h/mL (*p* < 0.0001)	[16]
Nanostructured Lipid Carrier (NLC)	Curcumin	Emulsion-ultrasonication; H125 solid–liquid lipid mix (glyceryl dibehenate, glyceryl monooleate, phosphatidylcholine) + Tween 80	117.28 ± 1.32 nm; Zeta −14.14 ± 0.30 mV; PDI < 0.3	99.99% (near-theoretical max); drug loading 1.73%	Sustained: <55% released over 24 h (vs. >90% for free CUR); stable in SGF (4 h); size increased in SIF (117 → 197 nm by 4 h) due to bile salt/enzyme disruption	This study evaluated pharmacokinetics and intestinal lymphatic transport mechanism in healthy rats and mice only. No diabetic model, no glycaemic endpoint, no antidiabetic claim can be attributed to this source	5.13-fold ↑ AUC0–t; 5.25-fold ↑ Cmax; 2.23-fold ↑ t1/2 (33.49 h) vs. free CUR solution, healthy rats (5 mg/kg). Direct lymphatic transport confirmed (CUR detected only in CUR-NLC group lymph; chylomicrons visualised by TEM)	[17]
Self-Microemulsifying Drug Delivery System (SMEDDS)	Berberine Hydrochloride (BH)	Microemulsification; 55% Capmul MCM (oil) + 22.5% Kolliphor RH40 (surfactant) + 22.5% 1,2-propanediol (co-surfactant); BH loading 20 mg/g	47.2 ± 0.10 nm; PDI 0.262 ± 0.006; self-emulsification 26.02 ± 0.24 s	Not reported as EE%; drug-loading/precipitation threshold used instead (stable ≤ 20 mg/g, precipitates ≥ 22 mg/g)	SGF (pH 1.2): 45.1 ± 1.7% at 300 min; SIF (pH 6.8): 93.1 ± 2.3% at 300 min (both > commercial tablets). No Caco-2 cytotoxicity at 50–600 µg/mL (viability > 95%)	This study evaluated pharmacokinetics in healthy rats only, benchmarked against commercial BH tablets. No diabetic model, no glycaemic endpoint, no antidiabetic claim can be attributed to this source	1.63-fold ↑ relative bioavailability (AUC0–∞); Cmax ↑ 2.09-fold (0.55 → 1.15 µg/mL); AUC0–∞ 5.116 ± 0.829 vs. 3.138 ± 0.263 µg·h/mL; t1/2 5.021 → 9.347 h	[18]
Nanoemulsion (Que-NE)	Quercetin	High-shear homogenisation + ultrasonication; ethyl oleate (oil) + Tween 20 + Labrasol (Smix); Box–Behnken optimised (9% Smix, 25% amplitude, 2.5 min)	Predicted 125.51 nm (exptl range 120.9–199.5 nm); PDI 0.215; Zeta −17.10 ± 5.61 mV	Predicted 87.04% (exptl range 60.16–91.08%)	Superior release vs. Que-PD (qualitative); stable ≤45 days at 30 °C storage, PDI ≤ 0.3 throughout	STZ-induced Type I diabetes, Wistar rats (note: Type I model, not T2DM), 12.5 mg/kg, 21 days. Significantly ↓ BGL, inhibited weight loss, improved OGTT, normalised lipid profile (TC/TG/LDL/VLDL/HDL), ↓ AST/ALT/BUN/creatinine, preserved islet/hepatic histology-pretreatment group (Que-NE(P)) best overall	4.46-fold ↑ AUC0–t; 5.32-fold ↑ AUC0–∞; 3.64-fold ↑ Cmax vs. Que-PD (healthy rats, 50 mg/kg). MRT 46.13 vs. 28.78 h	[69]
Self-Nanoemulsifying Drug Delivery System (SNEDDS)	Thymoquinone (TQ)	Microemulsification; Labrafil M2125 (oil) + Tween 80:Plurol oleique 3:1 (Smix); optimised F9 (30% oil, 50% Smix, 20% water); solid-SNEDDS on Avicel	90 ± 2.65 nm; Zeta −11.35 mV; PDI 0.312	Not reported as EE%; thermodynamic stability (centrifugation/heating-cooling/freeze–thaw: all Pass), 97.6% transmittance, XRD/DSC confirm amorphous entrapment	~80% released over 12 h (Higuchi model, r^2^ = 0.9834) vs. ~50% for pure TQ	This study evaluated paracetamol (PCM)-induced hepatotoxicity (liver injury model), not diabetes. TQ-SNEDDS significantly ↓ ALT/AST/ALP/bilirubin vs. TQ suspension; comparable/superior to silymarin; preserved liver histology. No diabetic model, no glycaemic endpoint, no antidiabetic claim can be attributed to this source	4-fold ↑ bioavailability (401.3% relative F) vs. TQ suspension, healthy rats (20 mg/kg). Cmax 98.92 vs. 28.34 µg/mL; AUC0–∞ 980.73 vs. 241.22 µg·h/mL	[75]

Note: this table reports verified physicochemical and pharmacokinetic characterisation data only. The in vivo models used to generate the bioavailability data reported here include cancer cell lines (curcumin-SLN), healthy-rodent pharmacokinetic studies (curcumin-NLC, berberine-hydrochloride SMEDDS, thymoquinone-SNEDDS), and a paracetamol-induced hepatotoxicity model (thymoquinone-SNEDDS), none of which involved a diabetic animal model. Abbreviations: ↑, increase/upregulation; ↓, decrease/downregulation; AUC, area under the plasma concentration–time curve; Cmax, maximum plasma concentration; DPP-4, dipeptidyl peptidase-4; EGCG, epigallocatechin gallate; EE, encapsulation efficiency; FBG, fasting blood glucose; G6Pase, glucose-6-phosphatase; GI, gastrointestinal; HbA1c, glycated haemoglobin; HFD, high-fat diet; LA:GA, lactide:glycolide ratio; MPS, mononuclear phagocyte system; MW, molecular weight; NF-κB, nuclear factor kappa-B; NLC, nanostructured lipid carrier; NLRP3, NLR family pyrin domain-containing protein 3; Nrf2, nuclear factor erythroid 2-related factor 2; PEPCK, phosphoenolpyruvate carboxykinase; P-gp, P-glycoprotein; PLGA, poly(lactic-co-glycolic acid); PEG, polyethylene glycol; RES, reticuloendothelial system; SIRT1, sirtuin-1; SLN, solid lipid nanoparticle; STZ, streptozotocin; t½, elimination half-life; TNF-α, tumour necrosis factor-alpha; TPP, tripolyphosphate; W/O/W, water-in-oil-in-water.

This self-emulsification behaviour, which leads to the reduction in the interfacial tension to near zero, results in the dissolution rate of the capillaries and thus in the rapid presentation of the drug in a solubilised state directly into the gastrointestinal lumen, which avoids dissolution rate-limited presentation [76,77]. In preclinical studies, the SNEDDS formulations of berberine, piperine and silymarin have been shown to increase oral bioavailability by two to eight times compared to conventional formulations with a simultaneous increase in the antihyperglycaemic activity [18,75,78]. Other formulation modifications to further enhance the stability and patient acceptance of the liquid SNEDDS are achieved by converting the liquid to a solid form via spray drying or adsorption onto inert carriers [73]. The concept of “phytosomes” is a totally “fresh” approach to drug delivery that employs covalently independent complexation of molecules, in which the phytochemicals are covalently linked to phospholipids, usually phosphatidylcholine, via non-covalent interactions, thus producing a complex with increased affinity for membranes. In contrast to classical encapsulation inside a nanocarrier, phytosomes are complexes formed by direct molecular association of the phytochemical with the phospholipid headgroup, where the phytochemical is complexed with a lipid-compatible complex that shows intestinal membrane permeability which is significantly higher than that of the uncomplexed phytochemical. The enhanced lipophilicity of the phytosome complex enhances passive transcellular absorption through the brush border membrane of the enterocyte, greatly increasing systemic bioavailability. The oral bioavailability and clinical anti-inflammatory and hepatoprotective properties of commercial phytosome–casein preparations of curcumin (Meriva^®^) and of silymarin (Siliphos^®^) have proven to be considerably better than their natural forms and thus support their strong potential for translation to new antidiabetic phytochemicals [61,72,76]. In total, nanoemulsions, SNEDDS, and phytosomes have a significant place in the phytochemical delivery tools available for use in T2DM therapies, as each possesses unique biopharmaceutical advantages and is perceived as simple to formulate, regulated (as far as law documents are concerned), and meaningful.

A critical appraisal of available nanocarrier platforms reveals that no single system offers universal advantages across all phytochemical candidates in T2DM (Table 3). Lipid-based platforms, SLNs and NLCs, offer the highest oral bioavailability enhancement ratios (3–8-fold) through lymphatic absorption and carry the most tractable regulatory pathway owing to their GRAS (generally recognised as safe) excipient composition; however, their limited loading capacity for highly polar phytochemicals and susceptibility to polymorphic lipid transitions constraining long-term drug retention represent meaningful formulation challenges [15,16,17]. PLGA nanoparticles offer the most established regulatory precedent among polymeric carriers and the most predictable sustained-release kinetics relevant to chronic T2DM management, but acidic degradation microenvironments may compromise structurally sensitive phytochemicals such as EGCG and resveratrol, and manufacturing scale-up requires extensive comparability data [68]. Chitosan nanoparticles are uniquely advantaged for gut-directed phytochemical delivery through their mucoadhesive properties and tight junction modulation, particularly relevant for phytochemicals whose gut microbiota prebiotic effects depend on colonic bioavailability, but the absence of an approved chitosan nanomedicine and batch-to-batch molecular weight variability pose regulatory and manufacturing hurdles [66]. SNEDDS offers the highest bioavailability enhancement among all platforms through spontaneous GI nanoemulsification, making them particularly suitable for lipophilic phytochemicals requiring rapid postprandial absorption, but the high surfactant content required raises GI tolerability concerns at chronic exposure levels relevant to T2DM management [18,73]. Phytosomes, while demonstrating the most mature clinical evidence base (Meriva^®^ curcumin [79]), are phospholipid complexes rather than true nanoparticles and carry regulatory classification ambiguity between supplement and drug frameworks that limits their clinical development potential. Platform selection for any specific phytochemical–indication combination should therefore be guided by the compound’s physicochemical properties, the target absorption site (proximal intestine vs. colon), the required release kinetics (immediate vs. sustained), the available clinical evidence, and the intended regulatory pathway (Table 3).

## 7. Mechanistic Evidence and Translational Landscape: From Bench to Bedside

Effective therapeutic credibility for any drug candidate as an antidiabetic is based not only on empirical observations of lowering blood glucose, but on a coherent mechanism of action with respect to how the agent acts in the biological systems that control blood glucose. In the last two decades, there has been a significant and increasing base of experimental evidence that systematically characterised the mechanism of action involved in phytochemical activity in T2DM, ranging from receptor-level molecular interaction to intracellular signalling cascade and whole organism metabolic response. These data, gained through a progressively advanced series of in vitro models and a variety of in vivo experimental systems, have led to an enticing scientific foundation supporting the antidiabetic properties of bioactive phytochemicals, including curcumin, berberine, quercetin, resveratrol and epigallocatechin gallate [5,6,12,26,83,84]. In addition to these basic mechanisms, more studies have identified the applicability of phytochemicals across the entire therapeutic spectrum, which surpasses the metabolising action of glucose. New clues and evidence are making it increasingly clear that the gut microbiome and the regulatory systems that control fat, genetic, and epigenetic changes are significant players in phytochemical activity, and could introduce a whole new box of ideas regarding mechanistic understanding, bringing T2DM to the forefront of being more than just a metabolic syndrome disease. The microbiota, as a metabolically active organ, dynamically reacts to dietary phytochemicals, which subsequently leads to dynamic changes in the gut microbial composition and function, which have been associated with an improvement in insulin sensitivity, inflammation and gut barrier integrity. At the same time, phytochemicals have demonstrated effects on epigenetic mechanisms, such as DNA methylation, histone modifications and other non-coding RNAs that can alter the metabolic transcriptional programme of insulin-sensitive tissues in ways that may lead to long-lasting metabolic benefits beyond the period of active treatment [85,86,87,88,89,90]. Although all these mechanistic and preclinical data are thus available, the conversion of these phytochemical therapies into clinically effective therapies has been difficult and, for the most part, still not done. It has not been a smooth, direct journey from an intriguing in vitro observation to a proven place in the clinic, and it is not surprising that the T2DM drug development pipeline has seen several compounds that have shown excellent preclinical efficacy in animal models but not so much in humans. Relative to other phytochemicals, however, the pharmacokinetics of these compounds, discussed in the previous section, represent a significant obstacle to their use, along with the variability of existing clinical formulations and dosing regimens, and the relative lack of well-designed, sufficiently powered randomised controlled trials [7,41,58,79,91,92,93]. This section reviews the currently available mechanistic and translational evidence of phytochemicals in T2DM from a molecular perspective and moves out to the clinical world. This section starts with a review of the most significant in vitro and in vivo mechanistic studies that have revealed the importance of phytochemicals in glycaemic regulation, insulin sensitisation and maintenance of pancreatic beta-cell mass and function. It then reviews how knowledge of gut microbiota modulation and epigenetic reprogramming is emerging as additional layers to the antidiabetic mechanism. Lastly current clinical evidence, the literature search for relevant ongoing/completed trials, and the determination of the primary translational gaps which could pave the way to realise the potential of phytochemical-based nanomedicines for the complete evidence-based management of T2DM (Table 4) [5,6,26].

### 7.1. In Vitro and In Vivo Mechanistic Studies: Glycaemic Control, Insulin Sensitisation and Beta-Cell Protection

Phytochemical studies related to this type of diabetes have been continued with varying experimental models including isolated enzyme-based and cell-based in vitro assays, genetically engineered rodent models and models of diet-induced obesity. These studies have identified several pathways that suggest pleiotropic interaction with the pathophysiological network responsible for the development of T2DM that underpins the multiple, often converging molecular mechanisms by which the antidiabetic action of phytochemicals is affected [26,27,83,84].

Quercetin, luteolin, and EGCG competitively inhibit α-glucosidase with IC_50_ values comparable to or lower than acarbose, confirmed in systematic enzymatic studies [83,84]. Phytochemicals additionally upregulate GLUT4 translocation in skeletal muscle and adipose tissue via PI3K/Akt signalling [44,45,46]. Berberine has been extensively used in this context, with several studies showing its activation of AMPK in the hepatocyte, the skeletal muscle cell and the adipocyte, by inhibition of the mitochondrial complex 1 and increasing the ratio between AMP and ATP [27,34,94,95]. Curcumin and resveratrol also activate the AMPK pathway, to a certain extent, through different upstream actions, and both further activate the sirtuin-1 (SIRT1) and peroxisome proliferator-activated receptor gamma (PPAR-gamma) pathways that regulate mitochondrial biogenesis and adipogenic differentiation [44,47,57].

**Figure 5:**
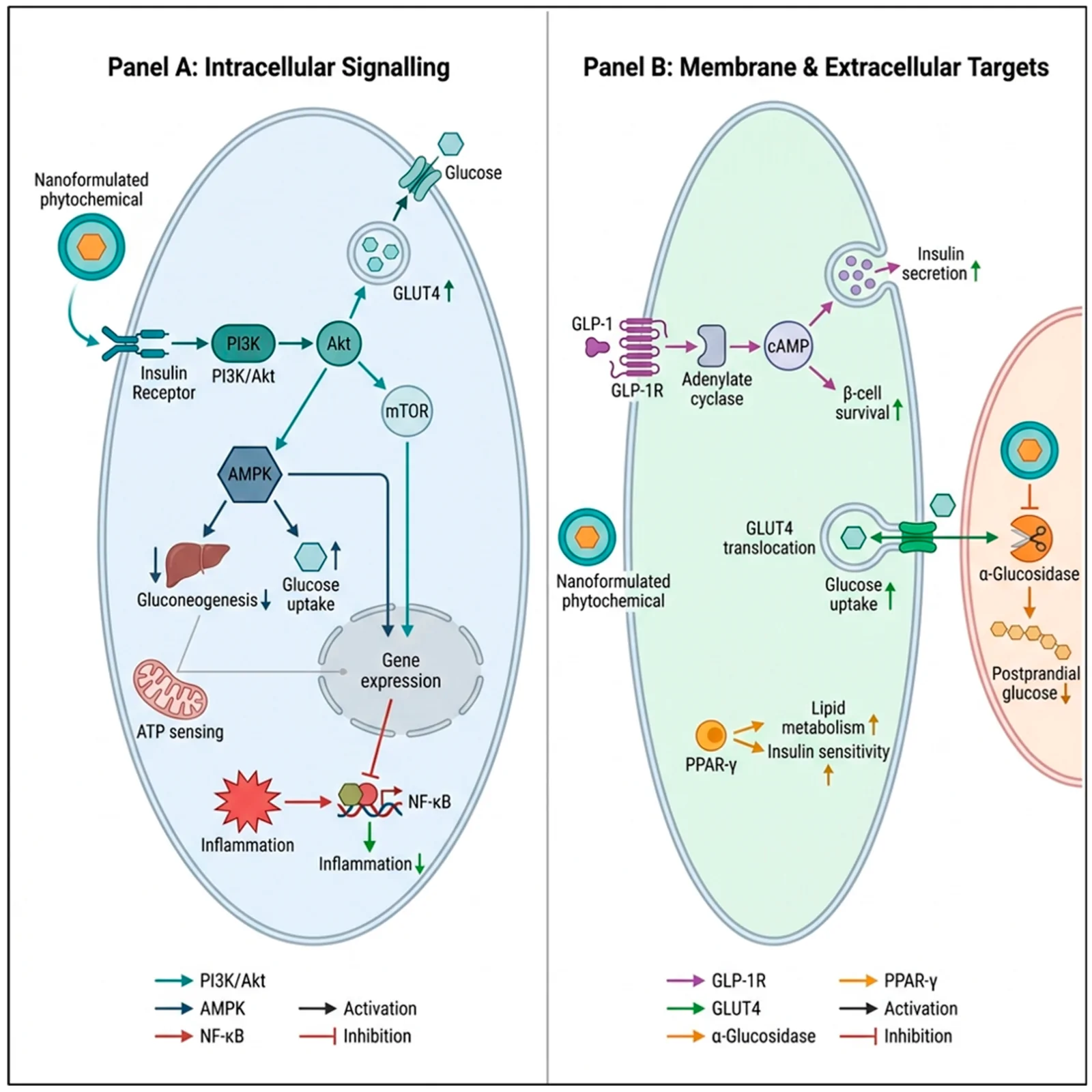
(Panel (**A**)) Intracellular signalling pathways modulated by nanoformulated phytochemicals. AMPK activation suppresses hepatic gluconeogenesis and promotes glucose uptake; PI3K/Akt activation facilitates GLUT4 translocation; NF-κB inhibition reduces chronic inflammation. Phytochemicals additionally modulate gene expression via AMPK/Akt nuclear signalling and regulate miR-375, miR-9, and miR-34a. (Panel (**B**)) Membrane-level and extracellular targets of nanoformulated phytochemicals. GLP-1R agonism promotes insulin secretion and β-cell survival; GLUT4 translocation enhances peripheral glucose uptake; α-glucosidase inhibition attenuates postprandial glucose absorption; PPAR-γ activation improves insulin sensitivity and lipid metabolism. Crosstalk between pathways (e.g., AMPK → GLUT4; PI3K/Akt → PPAR-γ) exists but is omitted for clarity. Abbreviations: AMPK, AMP-activated protein kinase; cAMP, cyclic AMP; GLP-1, glucagon-like peptide-1; GLP-1R, GLP-1 receptor; GLUT4, glucose transporter type 4; mTOR, mechanistic target of rapamycin; NF-κB, nuclear factor kappa-B; PI3K, phosphoinositide 3-kinase; PPAR-γ, peroxisome proliferator-activated receptor gamma.

As well as glycaemic control, the natural history of T2DM features continual loss of pancreatic beta-cell mass and secretory dysfunction attributed to continued glucolipotoxicity, endoplasmic reticulum stress, oxidative stress and inflammatory cytokine-mediated apoptosis. In models of beta-cells, phytochemicals have garnered remarkable cytoprotective activity, working via several complementary mechanisms. Curcumin inhibits the activation of inflammatory substances such as nuclear factor kappa-B (NF-κB) and reduces the pro-apoptotic protein expression of caspase-3 and Bax in cytokine-stimulated beta-cell lines. Quercetin is known to stimulate the nuclear factor erythroid 2-related factor 2 (Nrf2) transcription factor to induce the expression of antioxidant response element genes, including haem oxygenase-1 and superoxide dismutase, decreasing oxidative stress-induced beta-cell damage [33,47,89,90]. In diabetic rodent models, oral delivery of formulations containing nano-encapsulated phytochemicals has been correlated with the preservation of islet architecture, improvement in the density of insulin immunostaining, and improvement in glucose-stimulated insulin secretion in vivo, supporting a role for advanced delivery systems in optimising these in vivo protection effects at the tissue level with in vivo validation of the in vitro cytoprotective effects [12,15,17,61] (Figure 5).

### 7.2. Gut Microbiota Modulation and Epigenetic Reprogramming as Emerging Antidiabetic Mechanisms

A hallmark of these changes is the gut microbiome’s hyporeactivity to the benefits of short-chain fatty acid (SCFA)-producing bacteria and hyperactivity to inflammatory Gram-negative species, promoting a mechanism increasingly dubbed “microbial dysbiosis” [85,86,87], which falls under the category of antidiabetic mechanism research. At the same time, studies in epigenomics have shown that the metabolic phenotype of T2DM is not solely predicted by genetic predisposition but is significantly influenced by environmentally responsive epigenetic changes that regulate gene expression in insulin-sensitive tissues. Bioactive dietary components, namely phytochemicals, have come to be known for engaging both facets, making them candidate agents with multidimensional capabilities to influence the biological environment of T2DM at a depth unsuspected until recently, and beyond the immediate action on a specific target. The metabolites of gut microbiota, in particular short-chain fatty acids, such as acetate, propionate and butyrate generated by fermentation of dietary fibre and polyphenols, are known to have extensive systemic metabolic activities. They can be used as signalling molecules at G protein-coupled receptors on enteroendocrine cells to stimulate glucagon-like peptide-1 (GLP-1) and peptide YY, which inhibit appetite and promote insulin release and moderation of gastric emptying [96,97]. Several microbially active polyphenols found in the diet, such as resveratrol (RSV), quercetin (QU) and ellagic acid (EA), have been shown to selectively promote growth of well-known beneficial microbial groups (most notably *Akkermansia muciniphila* and *Lactobacillus* species), as well as inhibit the abundance of pro-inflammatory microbial groups (Firmicutes and lipopolysaccharide-producing Bacteroidetes) [86,97]. A specific interest has centred on *Akkermansia muciniphila*, which negatively correlates with BMI, systemic inflammation and insulin resistance in human cohort studies, and it has also been shown to be enriched due to polyphenol supplementation and associated with restoration of intestinal barrier integrity, as upregulation of tight junction proteins such as claudin-3 and occludin has been found [87,98]. Importantly, the prebiotic-like activity of phytochemicals may be enhanced when administered in a nanoencapsulated form, due to alteration in their colonic bioavailability and interaction kinetics with the prevailing bacterial populations in the distal colon [61,78].

Another layer of phytochemical action in T2DM studies is through epigenetic mechanisms, such as heritable changes in gene expression without changes to the DNA sequence. In T2DM patients, hypermethylation of various metabolic gene promoters, which silences the expression of key molecules (e.g., insulin receptor substrate-1 and GLUT4), has been identified in their tissues [88,98]. DNA methyltransferase enzymes were shown to be inhibited by curcumin, and it also modulated the activity of histone deacetylase enzymes, which can help reverse pathological hypermethylation patterns and restore transcriptional competence in metabolic genes [33,90,98]. Resveratrol activates the NAD-dependent deacetylase SIRT1, which deacetylates and thereby activates peroxisome proliferator-activated receptor gamma coactivator 1-alpha (PGC-1α), a master regulator of mitochondrial biogenesis and oxidative metabolism that is impaired in insulin-resistant skeletal muscle [44,46,57]. From the perspective of non-coding RNA, other phytochemicals have been shown to regulate the expression of microRNAs that are known to play a role in beta-cell function and insulin-signalling, such as miR-375, miR-9, and miR-34a, indicating that these compounds could also exert durable metabolic reprogramming [88,98,99]. The identification of these epigenetic mechanisms as true targets of phytochemical activity significantly expands the account of phytochemical action and highlights the strong arguments for studying the long-term metabolic consequences of chronic intake of high levels of phytochemicals in susceptible individuals (Figure 4).

### 7.3. Clinical Evidence, Ongoing Trials, and Translational Gaps

The clinical translation of mechanistic and preclinical data is the toughest and least achieved part of the phytochemical-T2DM research agenda so far. Although the existing in vitro, animal, and human data regarding the antidiabetic potential of these key phytochemicals are well documented and consistent, many challenges lie in the clinical study of botanical products and their formulations in humans, reflecting the diversity of available human data [85,86]. Of the phytochemicals with the strongest clinical evidence, curcumin has been studied in a few randomised controlled trials in patients with T2DM or prediabetes for 8–12 weeks, indicating, albeit relatively, significant improvements in fasting blood glucose, glycated haemoglobin (HbA1c) and insulin resistance [41,79,93]. One of the most impressive human examples of the antidiabetic properties of phytochemicals, a landmark 9-month randomised study of curcuminoid supplementation in a Thai prediabetic population demonstrated that curcuminoid supplementation significantly decreased the rate of progression to T2DM compared with placebo [79]. In fact, the clinical evidence base for berberine is quite extensive when compared with other phytochemicals, as several RCTs conducted in people with T2DM in China demonstrated reductions in fasting and postprandial glucose, HbA1c, total cholesterol (TC) and triglycerides that were similar to those achieved by comparable durations of treatment with metformin [58,91,92]. For resveratrol and quercetin, somewhat more complex effects have also been reported at the clinical level that do not align with in vivo studies, and this is often believed to be related to their extremely low oral bioavailability when administered in classic dietary supplements [57,59].

One of the key clinical translation milestones is the pilot trial of nanoformulations or advanced formulations instead of natural extracts or powders. Early-phase clinical trials have demonstrated a significantly elevated plasma-level concentration of curcumin in early-phase clinical trials with phospholipid-complexed curcumin (Meriva^®^) and a more pronounced anti-inflammatory effect, compared to standard forms of curcumin, indicating that delivery system innovation could be a key element in the achievement of clinical effectiveness [41,79,93]. There are a couple of long-term registered trials evaluating nanoemulsion and phytosome formulation curcumin, berberine and quercetin done in T2DM and metabolic syndrome patients to see reduction in HbA1c, insulin sensitivity indices, and inflammatory biomarkers as primary endpoints [61,76]. The results from these experimental trials will be eagerly awaited for their ability to offer the most direct available evidence of whether the pharmacokinetic benefits of nano-delivery systems seen in preclinical models can in fact be shown to translate into meaningful clinical benefits. Though all the above are encouraging, several gaps between translation in the real world and in the lab still exist and should be specifically detailed and addressed. Small-sized and short-duration clinical trials, heterogeneous patient populations, different formulations, and varying outcome measures are all problems, as these limit the interpretability and generalizability of clinical trial results published in the literature. Second, and more importantly, many clinical studies have not been characterised pharmacokinetically, which makes it challenging to define dose–response relationships and impossible to tie clinical effects to the introduced phytochemical rather than to confounding dietary or lifestyle factor(s). Third, the chronic exposure data for these nano-formulations in humans are incomplete, specifically relating to the possible build-up of the nanocarriers and their impact on immune responses, hepatic metabolism and gut microbial ecology. Fourthly, regulatory protocols for nano-phytochemical products are in a grey zone between traditional dietary supplements and new pharmaceutical products, leaving unclear the possible development path for their clinical use as well as the necessary level of evidence for authorisation [61,86]. Collaboration between formulation scientists, clinical pharmacologists, regulatory scientists, and health economists will be essential to cross the ‘Valley of Death’ between basic research and clinical trials, while the design and execution of properly powered, controlled Phase II and Phase III clinical trials, implemented with well-characterised and standardised nano-phytochemical preparations, will be required.

**Table 4:** Summary of clinical, preclinical, and epidemiological evidence for phytochemical and nanoformulation interventions in type 2 diabetes mellitus (T2DM), detailing study design, endpoints, outcomes, and limitations. (**A**) Clinical trials of phytosomal/nanoformulated curcumin; (**B**) conventional phytochemical extract randomised controlled trials, included as benchmark comparators for nanoformulation efficacy; (**C**) preclinical nanoformulation studies in animal models; (**D**) epidemiological cohort evidence linking phytochemical exposure to T2DM incidence.

(**A**)							
**Formulation**	**Study Design**	**Human n/Duration**	**Primary Endpoints**	**Key Outcomes**	**Mechanistic Basis**	**Limitations**	**Primary Source**
Phytosomal curcumin (Meriva^®^-phospholipid complex	RCT, double-blind, placebo-controlled; Thai prediabetic population	n = 240; 9 months	Rate of T2DM progression; FBG; HbA1c; HOMA-IR	0% T2DM progression in curcumin group vs. 16.4% in placebo; improved HOMA-β, HOMA-IR, adiponectin ↑	AMPK/PI3K-Akt ↑; NF-κB ↓; β-cell cytoprotection [57,58]	Single ethnic cohort (Thai); phospholipid complex ≠ nanoparticle; no PK arm in all subjects	Chuengsamarn S. *Diabetes Care* 2012; 35(11): 2121–2127. doi: 10.2337/dc12-0116 [91]
Phytosomal curcumin (250 mg/day oral)	RCT, double-blind, placebo-controlled; migraine patients (proxy for phytosomal safety/formulation performance)	n = 70; 8 weeks	Severity/frequency/duration of migraine; stress; sleep quality	Significant reduction in migraine attacks, stress scores, improved QoL and sleep quality; no adverse effects reported	Anti-inflammatory and neuroprotective via NF-κB ↓ [76]	Not a T2DM-specific trial; applied here for phytosomal curcumin formulation performance validation	Shojaei M et al. *Chin J Integr Med* 2025; 31(11): 963–972. doi: 10.1007/s11655-025-3939-1 [76] IRCT20201129049534N2
(**B**)							
CATEGORY II: Conventional phytochemical extract RCTs (benchmark data for nanoformulation comparators)							
**Phytochemical/Formulation**	**Study Design**	**Human n/Duration**	**Primary Endpoints**	**Key Outcomes**	**Mechanistic Basis**	**Limitations**	**Primary Source**
Berberine (0.5 g × 3/day oral) vs. metformin	RCT; newly diagnosed T2DM; Chinese population; active comparator: metformin	Study A: n = 36; Study B: n = 48; 3 months each	FBG; PPG; HbA1c; TG; LDL; HOMA-IR	HbA1c ↓ 9.5% → 7.5% (Study A); FBG ↓, PPG ↓, HOMA-IR ↓ 44.7%; effects comparable to metformin	AMPK via Complex I ↓; PEPCK/G6Pase ↓; GLUT4 ↑ [34,94,95]	GI adverse events in 34.5% of patients; no nanocarrier; single-centre pilot	Yin J, Xing H, Ye J. *Metabolism* 2008; 57(5): 712–717. doi: 10.1016/j.metabol.2008.01.013 [92]
Berberine (1.0 g/day oral) vs. placebo	RCT, double-blind; T2DM with dyslipidaemia; n = 116; Chinese population	n = 116; 3 months	FBG; PPG; HbA1c; TG; LDL; GDR (insulin clamp)	FBG ↓ 7.0 → 5.6 mmol/L; HbA1c ↓ 7.5% → 6.6%; TG ↓; LDL ↓; all *p* < 0.0001	AMPK activation; hepatic gluconeogenesis ↓; GLUT4 ↑; insulin sensitivity ↑ [34,58,91]	Predominantly Chinese cohort; oral bioavailability 0.36–0.68% (P-gp efflux); no nanocarrier	Zhang Y et al. *J Clin Endocrinol Metab* 2008; 93(7): 2559–2565. doi: 10.1210/jc.2007-2404 [91]
Curcumin/turmeric (various doses and formulations)-umbrella meta-analysis of 59 RCTs	Umbrella meta-analysis; mixed T2DM and disturbed glycaemia populations	Total n from 59 RCTs; 8–24 weeks	FBS; HbA1c; HOMA-IR	FBS ↓ 4.60 mg/dL; HbA1c ↓ 0.32%; HOMA-IR ↓ 0.33; complementary therapy benefit demonstrated	Multi-target glycaemic modulation; AMPK ↑; NF-κB ↓; SIRT1 ↑ [41,47,57]	Heterogeneous formulations; dose variability; short-term follow-up dominates pooled data	Dehzad MJ et al. *Diabetes Metab Syndr* 2023; 17(10): 102855. doi: 10.1016/j.dsx.2023.102855 [41]
Curcumin (various formulations)-systematic review of 11 RCTs	Systematic review; T2DM patients only	From 11 RCTs; ≥12 weeks superior to <12 weeks	FBG; HbA1c; HOMA-IR	Significant FBG and HbA1c reductions in 8/11 and 7/11 studies respectively; HOMA-IR reduced in 3/5 studies; ≥12-week intervention most effective	Glucose homeostasis multi-target modulation [41,57,91]	Formulation heterogeneity across studies; short durations; no pharmacokinetic characterisation	Mahdavi A et al. *Adv Exp Med Biol* 2021; 1291: 139–149. doi: 10.1007/978-3-030-56153-6_8 [93]
Berberine-umbrella meta-analysis of multiple RCTs	Umbrella meta-analysis; T2DM and metabolic disorders	Pooled from multiple RCTs	FBG; HbA1c; HOMA-IR; IL-6; TNF-α; CRP	FBG ↓ (ES −0.65 to −0.77); HbA1c ↓ (ES −0.57); HOMA-IR ↓ (ES −0.71 to −1.04); anti-inflammatory effects confirmed	AMPK ↑; hepatic gluconeogenesis ↓; immune modulation [34,58,94,95]	Predominantly Chinese cohorts; formulation heterogeneity; short follow-up in most RCTs	Nazari A et al. *Clin Ther* 2023; 46(2): e64–e72. doi: 10.1016/j.clinthera.2023.10.019 [58]
Quercetin-umbrella meta-analysis (18 RCTs)	Umbrella meta-analysis; mixed populations	From 18 RCTs	Insulin; FBG; SBP; various cardiometabolic markers	Insulin level ↓ (WMD −1.07 μIU/mL); SBP ↓; FBG not significantly changed; lipid profiles variable	α-glucosidase ↓; DPP-4 ↓; SIRT1 ↑; PI3K/Akt ↑ [37,47,59]	FBG not significantly reduced in pooled analysis; insufficient long-term RCT data	Arabi SM et al. *Phytother Res* 2023; 37(11): 5080–5091. doi: 10.1002/ptr.7971 [59]
(**C**)							
CATEGORY III: Preclinical nanoformulation studies (animal models)							
**Formulation**	**Preclinical Model/n**	**Key Preclinical Outcomes**		**Mechanistic Basis**	**Translational Gap**		**Primary Source**
Curcumin-SLN (glyceryl monostearate matrix; ~193 nm; EE > 90%)	STZ-induced diabetic rats; n = 8–12/group	Significantly improved plasma PK vs. free curcumin suspension; FBG ↓; anti-inflammatory markers ↓; lymphatic uptake ↑		Lipid-mediated lymphatic absorption bypasses first-pass; AMPK ↑; NF-κB ↓ [15,62]	No human T2DM RCT data yet for this specific SLN formulation		Saka R et al. *Pharmaceuticals* 2025; 18(4): 412. doi: 10.3390/ph14010412 [15]
Berberine-SLN (chitosan-coated; ~4.2-fold bioavailability ↑)	Gestational diabetes mellitus rats; n = 6–10/group	Oral bioavailability ↑ ~4.2-fold vs. berberine suspension; FBG ↓; insulin sensitivity ↑; AMPK activation ↑		Chitosan mucoadhesion + lipid lymphatic absorption; P-gp efflux bypassed [16,34]	No registered human clinical trial; gestational diabetes model may not generalise to T2DM		Liu Y et al. *Exp Biol Med* 2025; 250: 10255. doi: 10.1177/15353702251002559 [16]
Curcumin-NLC (CUR-NLC; ~135 nm; double-catheterised rat lymph model)	Sprague-Dawley rats; n = 6/group	Higher plasma AUC vs. free curcumin; lymphatic transport confirmed; EE > 92%		Amorphous lipid structure ↑ payload; lymphatic uptake avoiding hepatic extraction [17,61]	No human data; lymph cannulation model does not reflect clinical oral delivery		Chi H et al. *Pharmaceutics* 2025; 17(11): 1484. doi: 10.3390/pharmaceutics17111484 [17]
Quercetin nanoemulsion (Que-NE; ~125 nm; Box–Behnken design)	STZ-induced diabetic rats; n = 8–10/group	Superior FBG ↓, lipid profile ↑, β-cell and hepatocyte histology preserved vs. free quercetin		Nanoemulsion ↑ surface area for absorption; α-glucosidase ↓; GLUT4 ↑; NF-κB ↓ [42,69]	No human T2DM RCT; no Phase I safety data for this quercetin NE formulation		Mahadev M et al. *Pharmaceuticals* 2022; 15(1): 70. doi: 10.3390/ph15010070 [69]
Berberine-SNEDDS (droplet <68 nm; ~3.2-fold bioavailability ↑)	Sprague-Dawley rats; n = 6–8/group	Spontaneous nanoemulsification in GI fluid confirmed; bioavailability ↑ 3.2-fold vs. berberine suspension		Self-emulsification bypasses dissolution limitation; P-gp efflux partly circumvented [18,73]	No human clinical data; no T2DM-specific in vivo efficacy arm		Chen X et al. *Pharmaceutics* 2024; 16(11): 1484. doi: 10.3390/pharmaceutics16111484 [18]
Thymoquinone-SNEDDS (<90 nm; 4-fold bioavailability ↑)	Paracetamol-induced hepatotoxicity rodent model; n = 6/group	Bioavailability ↑ 4-fold; hepatoprotective activity significantly ↑ vs. pure TQ; rapid self-emulsification confirmed		Near-zero interfacial tension ensures GI solubilisation; lipid absorption pathway [75]	Not a T2DM model; hepatoprotective mechanism overlaps with antidiabetic but direct T2DM data absent		Rathore C et al. *Drug Deliv Transl Res* 2022; 13(1): 292–307. doi: 10.1007/s13346-022-01193-8 [75]
(**D**)							
CATEGORY IV: Epidemiological evidence							
**Exposure**	**Design/Population**	**n**	**Endpoint**	**Key Findings**	**Limitations**		**Primary Source**
Green tea consumption (EGCG content)	Prospective cohort studies; Japanese and Asian populations	n > 1,076,311 from 19 cohort studies	T2DM incidence; FBG; postprandial glucose	Inverse association between green tea consumption (≥3 cups/day) and T2DM risk; consistent across multiple East Asian cohorts	Confounding by diet and lifestyle; variation in tea preparation and EGCG content; retrospective dietary assessment; conventional infusion nanoformulation		Li X et al. *Nutr Res* 2023; 118: 116–127. doi: 10.1016/j.nutres.2023.08.002 [100]

Abbreviations: ↑, increase/upregulation; ↓, decrease/downregulation; AMPK, AMP-activated protein kinase; AUC, area under the plasma concentration–time curve; Cmax, maximum plasma concentration; CRP, C-reactive protein; DPP-4, dipeptidyl peptidase-4; EGCG, epigallocatechin gallate; ER, endoplasmic reticulum; FBG, fasting blood glucose; GI, gastrointestinal; GLP-1, glucagon-like peptide-1; GLUT4, glucose transporter type 4; GSK-3β, glycogen synthase kinase-3β; HbA1c, glycated haemoglobin; HFD, high-fat diet; HOMA-IR, homeostatic model assessment of insulin resistance; IKKβ, inhibitor of κB kinase β; IL-6, interleukin-6; IRS-1, insulin receptor substrate-1; JNK, c-Jun N-terminal kinase; MDA, malondialdehyde; MRP2, multidrug resistance protein 2; NF-κB, nuclear factor kappa-B; NLC, nanostructured lipid carrier; NLRP3, NLR family pyrin domain-containing protein 3; NP, nanoparticle; Nrf2, nuclear factor erythroid 2-related factor 2; OGTT, oral glucose tolerance test; PEPCK, phosphoenolpyruvate carboxykinase; PGC-1α, peroxisome proliferator-activated receptor gamma coactivator 1-alpha; P-gp, P-glycoprotein; PK/PD, pharmacokinetic/pharmacodynamic; PLGA, poly(lactic-co-glycolic acid); PPG, postprandial glucose; RCT, randomised controlled trial; SGLT1, sodium-glucose co-transporter 1; SIRT1, sirtuin-1; SLN, solid lipid nanoparticle; SNEDDS, self-nanoemulsifying drug delivery system; SOD, superoxide dismutase; STZ, streptozotocin; TG, triglycerides; TNF-α, tumour necrosis factor-alpha.

## 8. Challenges, Regulatory Considerations, and Future Perspectives

### 8.1. Toxicological Profiling, Safety Concerns, and Herb–Drug Interactions

A toxicological dimension specific to nanoformulated phytochemicals that has received insufficient attention in the literature concerns the amplification of known compound-associated risks that occurs as a direct consequence of the bioavailability improvements nanoformulation is designed to achieve. When a nanocarrier increases the oral bioavailability of berberine from its baseline 0.36–0.68% to clinically relevant systemic concentrations, as has been demonstrated in preclinical pharmacokinetic models, the same pharmacokinetic gain that may produce therapeutic benefit simultaneously amplifies every safety risk associated with that compound at elevated plasma concentrations [82,99,101]. Berberine at high systemic exposure has been associated with QT interval prolongation in isolated cardiac preparations and inhibition of cytochrome P450 CYP2D6 and CYP3A4, raising clinically important concerns about drug–drug interaction potential with co-administered cardiovascular and antidiabetic agents in the polypharmacy-exposed T2DM population [102]. Curcumin at supraphysiological concentrations demonstrates paradoxical pro-oxidant activity and CYP1A2/CYP3A inhibition, while green tea EGCG has been documented in a primary clinical pharmacokinetic study to reduce nadolol bioavailability by 85% through intestinal OATP1A2 (organic anion-transporting polypeptide 1A2) inhibition [103]. The critical implication is that safety thresholds established for conventional phytochemical dietary supplementation, including the GRAS designation for curcumin, were determined based on poor bioavailability inherent to conventional prep preparations and should not be assumed to apply to nanoformulated versions designed specifically to overcome those bioavailability limitations. Dedicated preclinical toxicological evaluation of each specific nano-phytomedicine at doses achieving the target therapeutic plasma exposure including ICH-compliant repeated-dose subacute (28-day) and subchronic (90-day) oral toxicity studies with full haematological, biochemical, and histopathological assessment is therefore mandatory before any nano-phytomedicine progresses to human Phase I trials, and existing toxicological data for conventional phytochemical preparations cannot be extrapolated as a surrogate safety profile for their nanoformulated counterparts [82].

The issue of nanocarrier component accumulation following chronic oral administration represents an additional and currently incompletely characterised safety dimension. Repeated oral dosing with lipid-based nanocarriers results in progressive enrichment of carrier lipid and surfactant components within enterocytes, mesenteric lymph nodes, and organs of the reticuloendothelial system, particularly the liver and spleen, as nanoparticles undergo receptor-mediated or non-specific endocytosis by resident Kupffer cells and splenic macrophages [68,69,82]. Chitosan-based nanoparticles, while generally biocompatible at acute exposure, raise concerns regarding immunostimulatory potential and membrane disruption at the cationic surface charge densities required for mucoadhesive function [66,82].

The genotoxicological framework applicable to nanomaterials, including the comet assay, micronucleus testing, and Ames assay, should be incorporated as standard components of repeated-dose oral nanoparticle safety assessment, as established in a 28-day integrative in vivo nanotoxicological evaluation. Beyond carrier-related effects, pharmacodynamic interactions in T2DM polypharmacy represent a particularly important clinical risk dimension. The intrinsic glucose-lowering activity of berberine, quercetin, and bitter melon phytopeptides when delivered at the substantially elevated systemic exposures achievable through nanoencapsulation could produce additive or synergistic hypoglycaemic effects in patients concurrently receiving metformin, sulphonylureas, DPP-4 inhibitors, or SGLT-2 inhibitors, precipitating clinically significant hypoglycaemia particularly in elderly or renally impaired patients in whom glucose counter-regulatory responses may be attenuated [49,54,58]. These pharmacodynamic interaction risks are not hypothetical extrapolations: they are direct and predictable consequences of the bioavailability amplification that is the primary engineering objective of nano-phytomedicine development, and they must be proactively characterised in pharmacokinetic–pharmacodynamic interaction studies conducted prior to chronic human administration.

Herb–drug interactions represent a clinically important and frequently underappreciated safety dimension that acquires heightened relevance in the context of T2DM patients, who are typically maintained on complex, multi-drug therapeutic regimens encompassing antihyperglycaemic agents, antihypertensives, lipid-lowering drugs, and antiplatelet therapies [102,104,105]. The pharmacokinetic mechanisms underlying herb–drug interactions most commonly involve the modulation of cytochrome P450 (CYP) enzyme activity and drug transporter function, two systems that collectively govern the metabolism and disposition of a large proportion of clinically prescribed medicines. Systematic screening of medicinal plant species has confirmed that several antidiabetic phytochemicals exhibit significant inhibitory activity against CYP enzymes and drug efflux/uptake transporters [102,106,107,108]. Curcumin and quercetin have been shown to inhibit both CYP1A2 and CYP3A activity in primary hepatocyte and hepatic microsome models, modifying the metabolism of warfarin and raising legitimate concerns about drug exposure in T2DM patients receiving anticoagulants, statins, or calcium channel blockers. Berberine has been documented to interact with cardiovascular drugs, including antihypertensives, through CYP enzyme modulation and transporter-mediated mechanisms; given that berberine is frequently combined with conventional antidiabetic therapy, including metformin in T2DM management, systematic pharmacokinetic interaction studies are warranted [1,2,109,110,111,112]. Of the confirmed clinical interactions, the most extensively documented involves green tea catechins consumed at 700 mL/day which dramatically reduced the maximum plasma concentration (Cmax) and area under the plasma concentration–time curve (AUC) of the β-blocker nadolol by 85.3% and 85.0%, respectively, in a randomised clinical pharmacokinetic study of ten healthy subjects, through inhibition of intestinal OATP1A2-mediated drug uptake rather than P-glycoprotein. This interaction has direct clinical relevance in T2DM patients with hypertension or cardiac disease managed with β-blocker therapy and is particularly important in the context of EGCG nano-formulations which may achieve substantially higher systemic EGCG concentrations than conventional green tea consumption, potentially amplifying this interaction into the range of clinical significance at doses lower than those currently associated with the documented effect.systematises the available pharmacokinetic herb–drug interaction evidence for the phytochemicals reviewed in this article. Systematic characterisation of CYP inhibition potential, time-dependent inhibition, and transporter interaction profiling (OATP1A2, OCT2 (organic cation transporter 2), P-gp, BCRP (breast cancer resistance protein)) through standardised in vitro assays and, where indicated, dedicated human pharmacokinetic interaction studies, should be incorporated as mandatory components of the development programme for any nano-phytomedicine product intended for chronic co-administration with conventional antidiabetic or cardiovascular therapies [82,99,102].

Curcumin’s nanotechnology applications have been reviewed across multiple therapeutic domains, including metabolic and oncological diseases [111], and combined phytochemical formulations such as those incorporating fenugreek galactomannans, cinnamaldehyde, and berberine present multi-target antidiabetic profiles that complicate both efficacy attribution and adverse effect monitoring [112]. From the perspective of gut microbiome-directed safety evaluation, short-chain fatty acids generated by fermentation of dietary polyphenols have been shown to associate inversely with adiposity, HbA1c, and insulin resistance while positively correlating with preserved β-cell function and GLP-1 secretion in human cohort studies [113]; however, whether the altered colonic bioavailability of polyphenols delivered via nanoencapsulation modifies this SCFA-mediated benefit or disrupts established commensal microbial ecology has not been systematically examined. FODMAPs and polyphenol supplementation can significantly enrich *Akkermansia muciniphila* populations and modulate tight junction protein expression [114], but the impact of co-administered phytochemical nanocarriers on this prebiotic-like effect is unknown. The design of oral nano-formulations intended to survive gastric and intestinal digestion and reach colonic microbial populations intact, exemplified by self-assembled chitosan–insulin nanoparticle systems that rely on mucoadhesion and reversible tight junction opening [115], introduces additional variables for microbiome-directed safety evaluation.

Phytochemicals that modulate autophagy via LC3-mediated pathways, including curcumin, resveratrol, kaempferol, and piperine, exhibit dual roles in tumour promotion/suppression depending on cellular context and dose [81], a complexity that extends to the T2DM setting where autophagy dysregulation contributes to β-cell death and insulin resistance. The link between gut dysbiosis, insulin resistance, and NAFLD progression, in which polyphenol-rich herbal interventions can modulate the interaction between intestinal microbiota and bile acid metabolism to improve hepatic insulin sensitivity [116], further illustrates the multidimensional safety and efficacy landscape of phytochemical nanomedicines. Quercetin exerts broad anti-ageing, cardioprotective, hepatoprotective, and antidiabetic effects primarily through SIRT1 and SIRT6 activation pathways that regulate glucose utilisation, fatty acid oxidation, mitochondrial biogenesis, and genome stability [117]; however, the implications of sustained quercetin-mediated sirtuin modulation at concentrations achievable via nano-formulated delivery for long-term metabolic homeostasis require careful evaluation. Indian medicinal herbs used for T2DM management, including those with dual protective effects against diabetes and Alzheimer’s disease, face shared challenges of poor phytoconstituent physicochemical properties that have driven innovative nano-formulation strategies [118], including lipid-based, polymeric, and phytosomal delivery systems. Berberine modulation of gut microbial ecology, specifically its metabolic conversion by gut bacteria into active metabolites, alteration of tight junction proteins, and regulation of tryptophan catabolism pathways [119], provides an additional mechanistic layer that must be characterised under nano-delivery conditions where berberine bioavailability and colonic residence time are substantially altered.

### 8.2. Regulatory Pathways, Standardisation Challenges, and Scale-Up

Nanomaterial delivery platforms designed to address pancreatic β-cell dysfunction, insulin resistance, and diabetic complications, including PLGA-based systems, solid lipid nanoparticles, liposomes, and niosome platforms, each present distinct manufacturing, characterisation, and regulatory challenges [120]. Phytoceutical nanoparticle delivery systems applied across oncological and metabolic disease contexts have validated the concept that SLN and liposomal platforms can substantially improve phytoceutical bioavailability and tumour/tissue targeting [121]; however, the regulatory pathway for approving such systems in T2DM, where the therapeutic bar requires demonstration of glycaemic, metabolic, and safety endpoints in long-term randomised controlled trials, is substantially more demanding. Resveratrol’s pleiotropic effects on obesity, insulin resistance, PPAR-γ, sirtuin activation, and gut microbiome composition make it an attractive nano-phytomedicine candidate [122]; however, translating these benefits from preclinical models to human RCTs has been hindered by extreme oral bioavailability variability. Tea consumption at ≥4 cups/day associates with a 17% reduced T2DM risk in meta-analyses spanning 19 cohort studies and >1 million participants [100], a finding that provides epidemiological support for EGCG-targeted nano-delivery but also highlights the challenge of translating population-level dietary signals into regulated nanomedicine products. Oral insulin delivery via polysaccharide-based nanocarriers, including pH-responsive chitosan systems with mucoadhesive properties that protect insulin from gastric enzymatic degradation and enhance paracellular intestinal uptake [123], exemplifies the type of advanced delivery innovation for which no clear regulatory pathway currently exists under either the drug product or dietary supplement framework.

Curcumin’s antioxidant properties, specifically its capacity to upregulate superoxide dismutase (SOD) through anti-inflammatory and neuroprotective mechanisms involving COX-2 suppression, NLRP3 inhibition, and NRF2/HO-1 (haem oxygenase-1) activation [124], represent mechanistic benefits relevant to both β-cell protection and diabetic complication prevention that must be evaluated as composite endpoints in regulatory submissions. Tetrahydropalmatine and structurally related phytochemicals that switch hepatic lipid metabolism via AMPK-SREBP-1c-SIRT1 signalling, ameliorating hepatic steatosis in NAFLD models relevant to T2DM comorbidity [125], illustrate the expanding mechanistic scope of phytochemical nano-medicines, but also the challenge of defining primary regulatory endpoints that capture multi-pathway therapeutic effects. Combined phytochemical formulations delivering resveratrol, EGCG, and ferulic acid synergistically enhance GLUT4 translocation and glucose uptake in insulin-resistant muscle and liver cells through both insulin-dependent (PI3K) and insulin-independent (AMPK, Ca^2+^) pathways [126]; however, the regulatory characterisation of such multi-component nano-platforms, including identification of the active pharmaceutical ingredient(s), dose–response relationships for each component, and demonstration of formulation stability, presents substantial analytical and regulatory complexity. Chrysin, despite potent antidiabetic, antioxidant, and anti-inflammatory properties, suffers from the same poor aqueous solubility and bioavailability that constrains most phytochemical candidates; PLGA, lipid-based, and micellar nanocarrier encapsulation of chrysin has demonstrated substantially improved pharmacokinetic profiles [127], but regulatory approval of such novel excipient combinations in diabetic populations requires demonstration of nanocarrier biocompatibility across the full spectrum of diabetic metabolic perturbations. Chinese herbal medicine preparations modulating gut microbiota composition, selectively enriching *Akkermansia*, *Bifidobacterium* and Firmicutes populations and reducing pathogenic species [128], provide a rich source of multi-phytochemical candidates for targeted nano-phytomedicine development, particularly where traditional clinical experience provides a foundation for safety characterisation.

The regulatory landscape governing nano-phytomedicines is characterised by substantial uncertainty across jurisdictions. No globally harmonised framework currently exists for nanomedicine products derived from botanical sources, and existing guidelines from the US FDA, EMA, and CDSCO address nano-drug products and herbal medicines through separate and often incompatible regulatory paradigms [99,102]. Medicinal plant extracts with cardiometabolic benefits, including those targeting multiple risk factors simultaneously, typically require assessment across glycaemic, lipid, inflammatory, and cardiovascular endpoints [129], a breadth that challenges the endpoint selection framework of standard regulatory submissions. Microbiome-targeted interventions including probiotics, prebiotics, synbiotics, and faecal microbiota transplantation have demonstrated significant reductions in body weight, BMI, lipid profiles, glucose metabolism, and inflammatory markers in obese and metabolic syndrome populations [130], providing a complementary evidence framework for nano-phytomedicines that target the gut-microbiome axis. Multi-phytochemical co-delivery platforms incorporating curcumin, resveratrol, quercetin, EGCG, and piperine exploit synergistic anticancer and anti-inflammatory mechanisms via co-delivery nanoparticle systems [131]; the regulatory characterisation of such combination products, including demonstration of pharmacological independence, absence of antagonistic interactions, and justification for each component’s inclusion, represents a significant analytical burden. The pharmacodynamic interaction between phytochemical nano-formulations and dual SGLT-1/SGLT-2 inhibitors such as sotagliflozin [132] must be systematically characterised, since additive inhibition of intestinal and renal glucose absorption could produce clinically significant hypoglycaemia in T2DM patients receiving both classes of treatment. Bioactive dipeptides and phenolic compounds that activate GLUT4 translocation via IGF-1R/IRS1/PI3K/Akt signalling, reducing adipose tissue lipid accumulation and hepatic steatosis [133], illustrate the expanding mechanistic repertoire of phytochemical candidates but also the challenge of demonstrating primary pharmacological action distinct from synergistic background dietary effects.

Phytochemical standardisation represents one of the most technically challenging problems in nano-phytomedicine product development. Plant biodiversity, exemplified by the extraordinary phytochemical richness of the Brazilian Cerrado, where multiple phenolics, flavonoids, and terpenoids with antidiabetic and anti-inflammatory activity exist across hundreds of endemic species [134], highlights both the opportunity and the complexity of sourcing reproducible phytochemical raw materials. Short-chain fatty acid profiles, including propionate and butyrate that associate with improved GLP-1 secretion, reduced HbA1c, and preserved β-cell function in human metabolic phenotype cohorts [135], provide quantifiable biomarkers that could inform both product standardisation specifications and clinical trial endpoint design for gut-directed nano-phytomedicine candidates. Ferulic acid, a phenylpropanoid with potent antidiabetic, antioxidant, and anti-inflammatory properties, demonstrates the challenge of clinical translation: despite robust preclinical evidence, poor bioavailability has necessitated diverse nanocarrier encapsulation strategies, and regulatory approval of these novel nano-ferulic acid preparations requires dedicated pharmacokinetic, safety, and efficacy characterisation [136]. TCM bioactive compounds including curcumin, gallic acid, and quercetin encapsulated in polymer nanoparticles, liposomes, SLNs, NLCs, and niosomes for prostate cancer and metabolic disease treatment demonstrate both the versatility of the nano-delivery toolkit and the formulation-specific pharmacokinetic profiles that complicate generalised regulatory frameworks [137]. Ginsenoside Rg3 and Rh2 prepared by biotransformation of protopanaxadiol-type ginsenosides with conversion rates approaching 98% at laboratory scale activate AMPK, NF-κB, MAPKs, and PI3K/Akt/mTOR pathways with antidiabetic, anti-inflammatory, immunomodulatory, and anticancer effects [138]; however, scaling this biotransformation process while maintaining chemical purity and bioactivity and managing the regulatory classification of biologically derived ginsenoside preparations represents a significant manufacturing science challenge.

### 8.3. Future Perspectives

TCM formulations that simultaneously modulate gut microbiota dysbiosis and bile acid metabolism preferentially enriching BSH (bile salt hydrolase)-active bacteria, accumulating ileal unconjugated bile acids, and upregulating intestinal FXR (farnesoid X receptor)/FGF15 (fibroblast growth factor 15) and TGR5 (Takeda G protein-coupled receptor 5)/GLP-1 signalling to improve glycaemia and insulin resistance [139] illustrate the multi-target potential of herbal nano-delivery approaches and provide a regulatory template where traditional clinical experience supports safety characterisation. Ginger (*Zingiber officinale*) bioactive compounds, including gingerols, shogaols, and zingerone, improve FBG, HbA1c (histone deacetylase), and insulin resistance through GLUT4 translocation, advanced glycation product inhibition, regulation of hepatic glucose metabolising enzymes, and cytokine modulation in clinical systematic reviews [140]. Gingerol encapsulation in nanoparticles, NLCs, nanoemulsions, and nanoliposomes enhances bioavailability and colonic delivery, with demonstrated enrichment of *Akkermansia* and *Muribaculaceae* and pathogenic species suppression in gut microbiome studies [141]. Repeated-dose oral nanoparticle safety evaluation, as demonstrated by the 28-day oral toxicity and genotoxicity assessment of silver-kaolin nanoparticle formulations establishing a NOAEL of 2000 mg/kg [142], provides a methodological template for the dedicated nanotoxicological characterisation that must accompany each new nano-phytomedicine candidate into regulatory submission. Curcumagalactomannoside (CGM) formulations using fenugreek galactomannan dietary fibre designed to deliver measurably higher free unconjugated curcuminoid plasma concentrations with superior tissue distribution compared with standard curcumin powder have been evaluated in 26 manuscripts including 12 clinical studies, establishing both pharmacokinetic performance and formulation-specific safety evidence [143].

*Moringa oleifera* bioactive compounds, including quercetin and kaempferol, exert anti-inflammatory effects through inhibition of cyclooxygenase, lipoxygenase, and NF-κB-mediated cytokine production, with documented activity against T2DM, NAFLD, hypertension, and cardiovascular disease [144]. The link between gut microbiota dysbiosis and diabetic microvascular complications, including the altered Firmicutes/Bacteroidetes ratio, reduced butyrate producers, and increased LPS-expressing species documented in diabetic retinopathy cohort studies [145], underscores the importance of evaluating nano-phytomedicine effects on diabetic complication trajectories as well as glycaemic endpoints. Natural products with NAFLD-targeting activity, including berberine and silymarin, both of which have advanced to Phase IV clinical trials and represent among the most clinically validated phytochemical candidates, modulate lipid metabolism, insulin resistance, oxidative stress, and FXR/PPAR-α targets [146]. Plant-derived essential oils and their bioactive constituents, including cinnamaldehyde, thymoquinone, eugenol, and linalool, modulate AMPK, GLUT4, NF-κB, SIRT1, PPAR-γ, and Nrf2/HO-1 signalling across systematic reviews of in vitro and in vivo antidiabetic evidence [147]. Flavonoid-rich nutraceutical combinations-curcumin, resveratrol, EGCG, quercetin, and luteolin-have demonstrated antioxidant, anti-inflammatory, antiviral, and immunomodulatory properties relevant to metabolic syndrome and T2DM management through SIRT1, NF-κB, and BDNF-mediated mechanisms [148].

**Figure 6:**
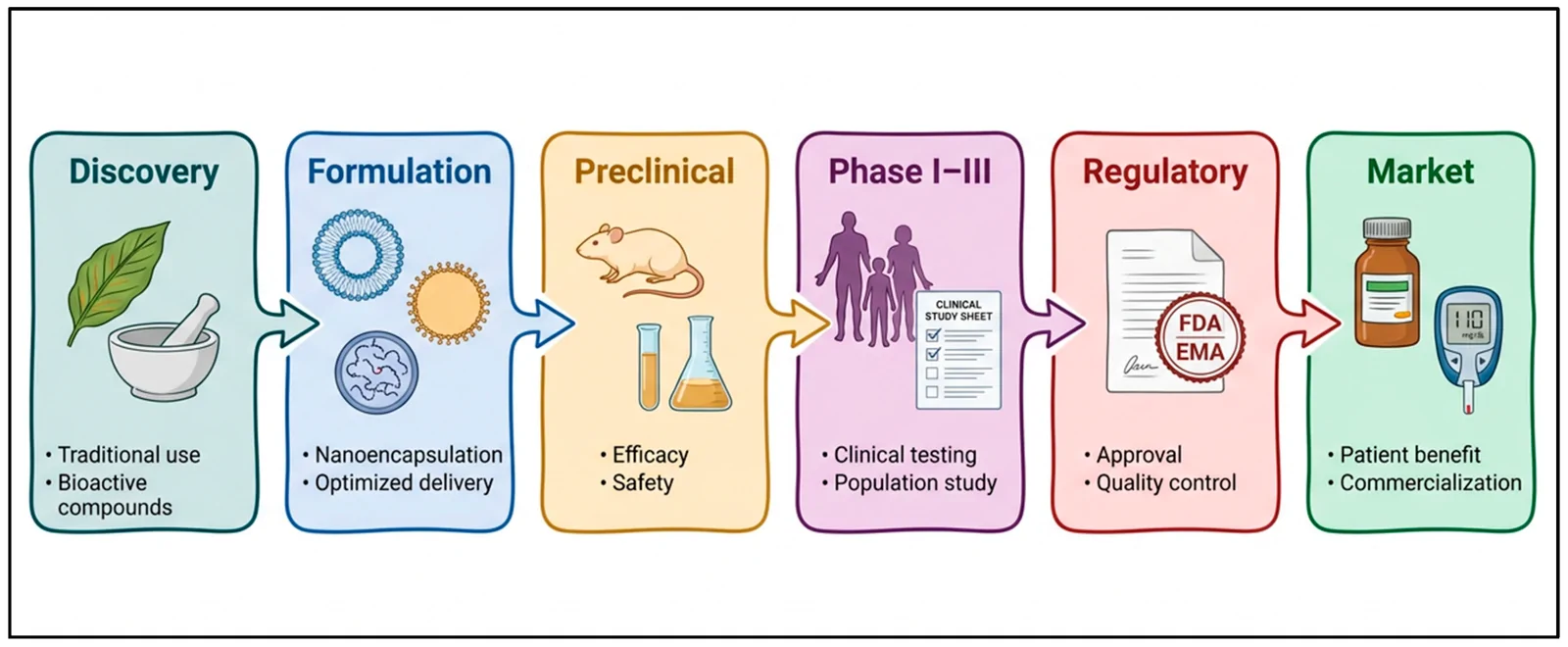
Translational roadmap: from ethnopharmacology to approved nano-phytomedicine in T2DM.

From the perspective of clinical evidence for herbal medicine combinations in T2DM, Gegen Qinlian decoction, in a systematic review and meta-analysis of 17 RCTs encompassing 1476 patients, significantly reduced FBG, 2hPG, HbA1c, total cholesterol, TG, LDL, and HOMA-IR, and improved HDL, when combined with conventional treatment [149]. Huanglian Jiedu decoction demonstrated significant improvements in HbA1c, FBG, 2hPG, HOMA-IR, and lipid profiles in a meta-analysis of 40 RCTs involving 3934 participants, establishing both efficacy and safety signals sufficient to support Phase II/III nano-formulation trial design [150]. Luteolin, a flavone that competitively inhibits α-glucosidase and α-amylase, demonstrates potent anti-inflammatory, epigenetic, and antidiabetic activity and has been formulated into nanoparticles, liposomes, nanoemulsions, microspheres, and hydrogels to address its unfavourable oral bioavailability, with ongoing clinical trials registered globally [151]. Nano-delivery systems for functional food bioactive compounds, including plant protein-based and polysaccharide-based carriers that improve bioactive stability, cellular uptake, and bioavailability, face shared challenges of safety evaluation, regulatory classification, and commercial-scale manufacturing consistency [152]. Pterostilbene, a naturally occurring dimethyl ether analogue of resveratrol with superior intestinal absorption, hepatic stability, and lower toxicity than stilbene counterparts, exerts antidiabetic effects through Nrf2-mediated pathways and has been studied in diverse formulations and clinical trials, serving as a promising candidate for nano-phytomedicine development with a more tractable pharmacokinetic profile than resveratrol itself [153] (Figure 6) (Table 5).

Phytochemicals present in citrus and grape sources, including hesperidin, rutin, naringin, quercetin, and procyanidin, consumed alongside vitamin D with probiotics and prebiotics nurture gut microbiota composition, suppress adipose-derived inflammatory cytokines including TNF-α, and enhance adiponectin to prevent metabolic syndrome and T2DM [154]. Anthocyanins are widely distributed bioactive polyphenols with hypoglycaemic activity mediated through GLUT4 translocation, DPP-IV inhibition, GLP-1 secretion promotion, PTP1B suppression, and SGLT-mediated glucose absorption delay, and represent an emerging class of phytochemical candidates for nano-encapsulated antidiabetic delivery [155]. Huanglian Wendan decoction demonstrated significant improvements in FBG, 2hPG, HbA1c, HOMA-IR, TC, TG, LDL-c, and HDL-c in a meta-analysis of 23 RCTs, with trial sequential analysis confirming sufficient information size to draw firm conclusions and no increased incidence of adverse events [156]. In the context of T2DM with carotid atherosclerosis, a common and clinically important comorbidity, combined Chinese herbal medicine has demonstrated significant improvements in carotid intima-media thickness, carotid plaque Crouse score, HbA1c, FBG, 2hPG, HOMA-IR, and β-cell function across a meta-analysis of 27 RCTs involving 2638 patients [157,158] (Table 4 and Table 5). Together, these clinical meta-analytic data from diverse herbal medicine traditions establish that well-characterised phytochemical preparations can achieve clinically meaningful glycaemic, lipid, and vascular endpoint improvements in T2DM populations, providing the evidence foundation that nano-formulation strategies seek to amplify and improve through enhanced bioavailability, targeted delivery, and mechanistic specificity.

Collaboration between formulation scientists, clinical pharmacologists, regulatory scientists, and health economists will be essential to cross the translational gap between basic research and clinical trials, while the design and execution of properly powered, controlled Phase II and Phase III clinical trials implemented with well-characterised and standardised nano-phytochemical preparations will be required to fulfil the considerable therapeutic potential of this field [82,99] (Table 6).

**Table 5:** Summary of clinical evidence quality for the antidiabetic effects of key phytochemicals: GRADE framework assessment.

Phytochemical	Best Available Human Evidence	No. Human Studies	Risk of Bias	Consistency	Directness	Precision	GRADE	Key Limitation
Curcumin	Umbrella meta-analysis 59 RCTs [41]; Systematic review 11 T2DM RCTs [93]; Prediabetes RCT n = 240, 9 months [79]	59 RCTs pooled [41]; 11 RCTs T2DM-only [93]; 1 landmark RCT [79]	Moderate-heterogeneous formulations; short durations dominate pooled data [41]	Moderate-consistent direction (FBG ↓ 4.60 mg/dL; HbA1c ↓ 0.32%; HOMA-IR ↓ 0.33) [41]; magnitude variable across formulations	Indirect-mostly conventional powder/extract; Meriva^®^ phospholipid complex ≠ true nanoparticle [79]; no true nano-curcumin RCT	Moderate small-to-modest effect sizes; ≥12 weeks more effective [93]	MODERATE	Formulation heterogeneity limits comparison; no standardised nano-curcumin human RCT
Berberine	Umbrella meta-analysis multiple RCTs [58]; Primary RCT n = 116 vs. placebo [91]; Primary RCT n = 36 + 48 vs. metformin [92]	>100 RCTs pooled [58]; 2 primary clinical trials [91,92]	Moderate-predominantly Chinese cohorts; heterogeneous formulations; short follow-up [58]	High-consistent FBG ↓, HbA1c ↓, TG ↓, LDL ↓, HOMA-IR ↓ across independent RCTs [58,91,92]	Indirect-conventional oral extract; oral bioavailability 0.36–0.68% (P-gp efflux); no nano-berberine RCT	High FBG ↓ ES −0.65 to −0.77; HbA1c ↓ ES −0.57; effects comparable to metformin [58,91,92]	MODERATE	Ethnic restriction (Chinese populations); no nanocarrier RCT; P-gp efflux limits exposure
Quercetin	Umbrella meta-analysis 18 RCTs [59]	18 RCTs pooled [59]	Moderate-variable dosing, aglycone vs. glycoside forms mixed; varied populations	Low-FBG not significantly changed in pooled analysis; insulin ↓ modest (WMD −1.07 μIU/mL); SBP ↓ only consistent finding [59]	Indirect-conventional supplement forms; no quercetin nanoparticle RCT	Low primary glycaemic endpoint (FBG) not significantly reduced; insufficient long-term data [59]	LOW	FBG not significantly improved in meta-analysis; P-gp efflux and hepatic conjugation reduce systemic exposure; no human nano-quercetin RCT
Resveratrol	Narrative review of small-scale human RCTs n = 19–75 [122]	Limited to small-scale RCTs only; no pooled human-only meta-analysis identified [122]	High-inter-individual variability in intestinal absorption and sulfation/glucuronidation [122]	Low-inconsistent results across trials; high variability in glycaemic outcomes [122]	Indirect-conventional supplement; no human nano-resveratrol RCT; SLN/PLGA preclinical only	Low modest, highly variable FBG/HbA1c changes; no powered RCT [122]	VERY LOW	Extreme oral bioavailability variability (rapid conjugation); no large RCT of any resveratrol formulation in T2DM; all nanoformulation data preclinical only
EGCG/Green Tea	Epidemiological cohort meta-analysis n > 1,076,311 [100]; Systematic review isolated EGCG RCTs (n = 20–60) [48]	>19 cohort studies n > 1,076,311 (epidemiology) [100]; RCTs n = 20–60 (isolated EGCG) [48]	Low risk (epidemiology) [100]; Moderate–High (isolated EGCG RCTs) [48]	Moderate (epidemiology: ≥4 cups/day → 17% T2DM risk ↓) [100]; Low (RCTs: inconsistent outcomes due to formulation heterogeneity) [48]	Very indirect epidemiology reflects dietary green tea consumption, not EGCG nanoformulation; isolated EGCG RCTs show inconsistent results [48]	Low for RCTs variable glycaemic endpoints; high endpoint heterogeneity across studies [48]	LOW	Chemical lability at alkaline GI pH (>80% degradation in free form); MRP2/P-gp efflux; epidemiological evidence does not translate directly to isolated EGCG or nanoformulation efficacy; no nano-EGCG human RCT
Gymnemic Acid	Systematic review *G. sylvestre* in obesity/diabetes [49]	Limited open-label studies only; specific n and duration unverifiable-removed from Table 3 [49]	Very high-no trial registration identified; no verifiable primary RCT source [49]	Not assessable-no reproducible RCT data	Indirect-conventional *G. sylvestre* extract; no nanoparticle clinical data [49]	Not assessable no quantitative pooled data available	VERY LOW	No verifiable primary RCT with trial registration; complex gymnemic acid isoform mixture complicates standardisation; nanoparticle clinical data entirely absent
Andrographolide	STZ-induced diabetic rodent models only [51]	0 verified human RCTs	Not applicable-preclinical evidence only [51]	Not applicable -STZ rodent model only [51]	Very indirect-STZ rodent model; no human data	Not applicable	VERY LOW	No verified human clinical trial or trial registration identified; all evidence preclinical (STZ-induced diabetic rats) [46]; low aqueous solubility constrains conventional delivery; nanoencapsulation promising but human data absent
TCM Combinations (berberine-containing)	Meta-analysis Gegen Qinlian 17 RCTs n = 1476 [149]; Meta-analysis Huanglian Jiedu 40 RCTs n = 3934 [144]; Meta-analysis Huanglian Wendan 23 RCTs [156]; Meta-analysis CHM + carotid atherosclerosis 27 RCTs n = 2638 [157]	>100 RCTs across four meta-analyses; total n > 12,000 [149,150,156,157]	Moderate-predominantly Chinese populations; risk of bias in smaller included RCTs; publication bias possible [149,150]	High-consistent FBG ↓, HbA1c ↓, 2Hpg (2 h postprandial glucose) ↓, HOMA-IR ↓, lipid improvements across multiple independent meta-analyses and formulations [149,150,156,157]	Indirect-multi-herb decoctions; active ingredient attribution to single phytochemical uncertain; conventional decoction; no nano-TCM RCT	High-large, pooled n; consistent effect direction and magnitude across multiple meta-analyses [149,150,156,157]	MODERATE	Ethnic restriction limits generalisability; individual phytochemical attribution unclear in multi-herb preparations; no nanoformulation RCT; predominantly Chinese regulatory/clinical context

Abbreviations: ↓, decrease/downregulation.

**Table 6:** Clinically relevant pharmacokinetic herb–drug interactions of key antidiabetic phytochemicals: evidence from in vitro and clinical studies.

Phytochemical	Target	Mechanism	Affected Drug(s)	Effect	Clinical Significance	Evidence
Curcumin	CYP1A2, CYP3A (inhibition)	Co-administration with warfarin decreased CYP1A2 and CYP3A protein expression and microsomal activity in porcine hepatocytes	Warfarin; any CYP1A2/CYP3A substrate	↑ drug exposure; modified warfarin metabolism	Potential bleeding risk; statin myopathy; calcium channel blocker hypotension in T2DM cardiovascular polypharmacy	In vitro (porcine primary hepatocytes + liver microsomes) [103]
Quercetin	CYP1A2, CYP3A (inhibition)	Decreased CYP1A2 and CYP3A activity confirmed in porcine hepatic microsomes; further enhanced by co-exposure with curcumin	Warfarin; statins; amlodipine	↑ plasma exposure of CYP substrates	Anticoagulation risk; statin myopathy; hypotension in T2DM polypharmacy	In vitro (porcine primary hepatocytes + liver microsomes) [103]
Berberine	CYP enzymes, P-glycoprotein	Competitive CYP inhibition and P-gp modulation; interactions with antihypertensive and cardiovascular drugs documented in comprehensive review	Antihypertensives; metformin (OCT2); cardiovascular drugs	↑ drug plasma levels; altered haemodynamic response	Berberine is routinely co-prescribed with metformin in T2DM; CYP and transporter interactions require monitoring	[102,159]
Green tea (EGCG)	OATP1A2 (inhibition)	Green tea (700 mL/day × 14 days) inhibited OATP1A2-mediated intestinal uptake of nadolol (IC_50_ = 1.36%); reduced OATP1A2-mediated nadolol uptake in HEK293 cells expressing OATP1A2	Nadolol (β-blocker); OATP1A2 substrates	↓ nadolol Cmax 85.3%; ↓ AUC_0–48_ 85.0% without altering renal clearance	Clinically documented-significantly reduced β-blocker antihypertensive effect in T2DM patients with cardiovascular comorbidity	Clinical RCT, n = 10 healthy subjects [103]
Ginseng/Ginsenosides	CYP enzymes, cardiovascular drug targets	Ginseng interacts with antihypertensives via CYP modulation and pharmacodynamic mechanisms	Antihypertensives; cardiovascular drugs	Variable drug exposure; haemodynamic effects	Monitor in T2DM patients on antihypertensive therapy receiving ginseng supplementation	[154]
Multiple phytochemicals	CYP2D6, CYP3A4, P-gp	Herbal medicinal plant screening against CYP enzymes and transporters (P-gp, BCRP, MRP2) using validated in vitro fluorometric assays	Multiple prescribed drugs	Variable inhibition across species and enzymes	Mandatory CYP/transporter profiling recommended for all antidiabetic phytochemical candidates	In vitro screening [102]

Abbreviations: ↑, increase/upregulation; ↓, decrease/downregulation.

## 9. Conclusions

The present review has synthesised mechanistic, preclinical, and clinical evidence for phytochemical-based nanomedicine in T2DM across four hierarchically distinct evidence tiers, each of which warrants careful interpretation with respect to the strength and clinical translatability of the supporting data. At the mechanistic level, a rigorous body of in vitro evidence has established that curcumin, berberine, quercetin, resveratrol, EGCG, and structurally related phytochemicals engage multiple therapeutically relevant molecular targets implicated in T2DM pathophysiology, including AMPK, PI3K/Akt/GLUT4, NF-κB, Nrf2/HO-1, α-glucosidase, DPP-4, and epigenetic regulatory axes governing metabolic gene expression in insulin-sensitive tissues. The convergence of these pleiotropic mechanisms across multiple pathological nodes provides a scientifically compelling rationale for phytochemical development, while simultaneously underscoring that in vitro mechanistic evidence does not constitute clinical proof of efficacy and that the concentrations required for target engagement in cell-based systems frequently exceed those achievable through conventional oral administration in vivo.

The biopharmaceutical rationale for nanoformulation is correspondingly well-founded. The poor aqueous solubility, extensive first-pass hepatic metabolism, P-glycoprotein efflux, and rapid phase II conjugation collectively restrict the oral bioavailability of most antidiabetic phytochemicals to below five per cent, representing formidable pharmacokinetic barriers to therapeutic utility. Preclinical pharmacokinetic studies have demonstrated that lipid-based and polymeric nanocarrier encapsulation substantially improves oral bioavailability parameters relative to free-drug comparators in rodent models, with enhancements ranging from approximately 1.6-fold for SMEDDS-formulated berberine to greater than 4-fold for quercetin nanoemulsion. In vivo antidiabetic efficacy studies in streptozotocin-induced diabetic rodents have further documented improvements in fasting blood glucose levels, islet architecture, and oxidative stress biomarkers across several nanoformulated candidates. These preclinical findings are promising; however, single-dose STZ insulinopenic models do not faithfully replicate the combined insulin resistance and progressive β-cell dysfunction of human T2DM, and bioavailability parameters from healthy rodent models may not predict outcomes in the altered metabolic milieu of diabetic human subjects.

The critical and incompletely bridged translational gap concerns human clinical evidence. Conventional, non-nanoformulated curcumin and berberine preparations have demonstrated moderate GRADE clinical evidence for glycaemic improvement, including HbA1c reductions of 0.32% and 0.9–1.2%, respectively, across pooled RCTs, and a landmark nine-month trial confirming reduced T2DM progression with curcuminoid supplementation in prediabetic adults. These findings are clinically meaningful but pertain exclusively to conventional preparations; they cannot be attributed to nanoformulation technology. No adequately powered, registered Phase II or III randomised controlled trial of a true nanoparticle phytomedicine in T2DM patients has been completed at the time of this review, and GRADE evidence for all nanoformulations therefore remains very low. Equally, no direct head-to-head comparative trial evaluating any nano-phytomedicine against approved first-line antidiabetic pharmacotherapies, metformin, SGLT-2 inhibitors, GLP-1 receptor agonists, or DPP-4 inhibitors has been registered or completed. Claims of superiority or therapeutic equivalence to established antidiabetic agents are accordingly not advanced in this review.

The appropriate clinical characterisation of nano-phytomedicines at the present stage is as investigational candidates with mechanistically credible antidiabetic activity, favourable preclinical pharmacokinetic profiles, and a supportive conventional clinical evidence base, but an unmet requirement for primary human pharmacokinetic and efficacy data. Realising their therapeutic potential will require Phase I human pharmacokinetic studies for the most advanced nanoformulated candidates, resolution of the regulatory classification ambiguity governing nano-botanical products under FDA, EMA, and CDSCO frameworks, and standardisation of phytochemical raw material quality and nanoparticle manufacturing to commercial-scale reproducibility. Only when these translational prerequisites are systematically addressed will it be possible to determine whether the considerable mechanistic promise and preclinical performance of nano-phytomedicines translate into a clinically validated contribution to evidence-based T2DM management.

The translational potential of nano-phytomedicines for T2DM management is scientifically credible and merits serious investment in clinical development. The priority actions identified by this review are (i) registration and execution of Phase I safety and human pharmacokinetic studies for the most advanced nanoformulated phytochemical candidates specifically nano-curcumin, nano-berberine, and nano-quercetin platforms with reproducible preclinical pharmacokinetic profiles; (ii) adoption of the GRADE evidence framework and five-category evidence separation model, as applied in this review, as standard for future phytomedicine review articles to prevent conflation of in vitro, animal, and clinical evidence; (iii) standardisation of phytochemical raw materials and nano-manufacturing processes to enable reproducible clinical-grade production; and (iv) prospective pharmacokinetic–pharmacodynamic modelling to define target plasma phytochemical concentrations needed to reproduce the mechanistic effects documented in vitro. Resolution of these translational gaps, rather than the mechanistic or formulation challenges, which are substantially addressed, represents the critical path to realising the therapeutic potential of nano-phytomedicines in T2DM management.

## Figures and Tables

**Table 3:** Comparative analysis of nanocarrier platforms for antidiabetic phytochemical delivery: advantages, limitations, and T2DM-specific suitability.

Platform	Oral Bioavailability Enhancement	Key Advantage	Key Limitation	T2DM Payload Suitability	Manufacturing Scalability	Regulatory Status	Best Matched Phytochemical(s)
Liposomes	Moderate (2–5-fold)	Phospholipid bilayer mimics cell membrane; excellent biocompatibility; both hydrophilic and hydrophobic payloads	Physical instability (oxidation, hydrolysis, leakage); high manufacturing cost; short shelf-life without lyophilisation	Moderate-β-cell targeting feasible; poor stability in GI environment without surface modification	Moderate-thin-film hydration at scale is challenging; microfluidics emerging [68]	Approved excipients; established regulatory precedent	Curcumin, EGCG [49]
SLNs	High (3–6-fold)	Solid lipid matrix protects cargo; lymphatic absorption enhances oral delivery; GRAS lipid excipients; relatively low toxicity	Burst release at body temperature (lipid polymorphic transition); drug expulsion on storage; limited loading capacity for highly lipophilic compounds	High lipid-mediated lymphatic absorption bypasses first-pass; AMPK activation preserved in vivo [15,16]	Moderate high-pressure homogenisation at scale feasible but energy-intensive	GRAS lipid excipients; minimal regulatory barriers	Curcumin [15], Berberine [16], EGCG [63]
NLCs	High (4–8-fold)	Amorphous lipid blend prevents drug expulsion; higher loading vs. SLN; better long-term stability	More complex formulation than SLN; excipient sourcing variability affects batch reproducibility	High superior to SLN for highly lipophilic phytochemicals; amorphous matrix prevents crystallisation	Moderate-like SLN but more formulation variables to control [17]	GRAS excipients; established lipid NP regulatory framework	Curcumin [17], EGCG
PLGA NPs	Moderate–High (3–6-fold)	Biodegradable; FDA-approved polymer; controlled sustained release; PEGylation reduces hepatic extraction	Acidic microenvironment from lactic/glycolic acid degradation may damage sensitive phytochemicals; burst release possible; batch reproducibility at scale challenging	High sustained release matches chronic T2DM treatment requirement; islet architecture preservation in preclinical models [68,80]	Moderate nanoprecipitation scale-up feasible; comparability data required for process changes [68]	FDA-approved polymer (PLGA); most established regulatory precedent among polymeric NPs	Curcumin [80], Berberine, Quercetin
Chitosan NPs	Moderate (2–4-fold)	Mucoadhesion prolongs GI residence; positive charge opens tight junctions; insulin and phytochemical co-delivery feasible [81]	pH-dependent solubility (insoluble at neutral/alkaline pH); batch-to-batch MW variability; cationic cytotoxicity at high concentrations [66,82]	Moderate mucoadhesion beneficial for colonic phytochemical delivery targeting gut microbiota; tight junction opening aids EGCG/quercetin paracellular transport	Moderate-ionic gelation scalable; MW standardisation critical	Generally recognised as safe, but the degree of deacetylation must be controlled; no approved chitosan nanomedicine yet	EGCG [48], Quercetin [69], Berberine, Insulin
Nanoemulsions (NE)	High (3–8-fold)	Thermodynamically/kinetically stable; high surface area; spontaneous or low-energy formation; transparent appearance	Surfactant toxicity at high concentrations; limited to lipophilic payloads; stability sensitive to dilution, temperature, and ionic strength	High rapid GI solubilisation particularly advantageous for poorly soluble phytochemicals (quercetin, curcumin) [70,74]	High microfluidisation and high-pressure homogenisation both scalable	Established excipients; NE-based products approved	Quercetin [70], Curcumin [74], Thymoquinone [75]
Phytosomes	Moderate–High (2–8-fold)	Phospholipid complexation improves lipophilicity AND membrane permeability; close to natural product origin; well-tolerated	Not a true nanoparticle; classified as phospholipid complex; regulatory ambiguity (supplement vs. drug); limited controlled release	Moderate clinical evidence exists for Meriva^®^ curcumin [79]; phytosomal form improves bioavailability at modest cost	High-solvent evaporation method scalable; commercially available platforms	Regulatory ambiguity: classified as supplement in most jurisdictions; drug classification requires clinical evidence	Curcumin [76], Quercetin, Silymarin
SNEDDS	Very High (3–10-fold)	Spontaneous nanoemulsification in GI fluid; no particle size degradation during storage (isotropic liquid system); rapid drug solubilisation; solid SNEDDS (spray-dried) addresses palatability	High surfactant content (20–60%) risks GI irritation and mucosal damage; liquid formulation palatability poor; drug-excipient compatibility critical	High rapid dissolution and absorption ideal for immediate postprandial glucose control (cinnamaldehyde, berberine, quercetin) [18,74,75]	High mixing technology scalable; solid SNEDDS via spray drying adds a step but is manageable	Established GRAS surfactant/co-surfactant excipients; SNEDDS regulatory pathway via drug NDA/ANDA	Berberine [18], Quercetin, Thymoquinone [75], Curcumin

## Data Availability

This article is a review of the previously published literature. No new datasets were generated or analysed during the preparation of this manuscript. All data discussed and referenced in this review are available within the cited published studies.

## Supplementary Materials

The following supporting information can be downloaded at https://www.mdpi.com/article/10.3390/pharmaceutics18091100/s1. Table S1. Comparative Scope Analysis of the Present Manuscript Against Representative Prior Reviews on Phytochemicals and Nanoformulation Strategies in Type 2 Diabetes Mellitus.

## Abbreviations

The following abbreviations are used in this manuscript:

ABCB1	ATP-binding cassette subfamily B member 1 (P-glycoprotein)
AMPK	AMP-activated protein kinase
ANDA	Abbreviated new drug application
AUC	Area under the plasma concentration–time curve
CDSCO	Central Drugs Standard Control Organisation (India)
Cmax	Maximum plasma concentration
COMT	Catechol-O-methyltransferase
CRP	C-reactive protein
CYP	Cytochrome P450
DPP-4	Dipeptidyl peptidase-4
EE	Encapsulation efficiency
EGCG	Epigallocatechin gallate
EMA	European Medicines Agency
ER	Endoplasmic reticulum
FBG	Fasting blood glucose
FDA	(U.S.) Food and Drug Administration
FD&C Act	Federal Food, Drug, and Cosmetic Act
FOXO1	Forkhead box protein O1
G6Pase	Glucose-6-phosphatase
GACP	Good agricultural and collection practices
GI	Gastrointestinal
GLP-1/GLP-1R	Glucagon-like peptide-1/Glucagon-like peptide-1 receptor
GLUT4	Glucose transporter type 4
GRAS	Generally recognised as safe
GSIS	Glucose-stimulated insulin secretion
GSK-3β	Glycogen synthase kinase-3β
HbA1c	Glycated haemoglobin
HBD	Hydrogen-bond donor
HDAC1	Histone deacetylase 1
HFD	High-fat diet
HMPC	Committee on Herbal Medicinal Products
HOMA-IR	Homeostatic model assessment of insulin resistance
IDF	International Diabetes Federation
IKKβ	Inhibitor of κB kinase β
IL-1β/IL-6	Interleukin-1 beta/Interleukin-6
IRS-1	Insulin receptor substrate-1
JNK	c-Jun N-terminal kinase
LA:GA	Lactide:glycolide ratio
MDA	Malondialdehyde
MHCP	Methylhydroxychalcone polymer
MPS	Mononuclear phagocyte system
MRP2	Multidrug resistance protein 2
MW	Molecular weight
NF-κB	Nuclear factor kappa-B
NLC	Nanostructured lipid carrier
NLRP3	NLR family pyrin domain-containing protein 3
Nrf2	Nuclear factor erythroid 2-related factor 2
OGTT	Oral glucose tolerance test
PDX-1/NKX6.1/MafA	β-cell identity transcription factors
PEG	Polyethylene glycol
PEPCK	Phosphoenolpyruvate carboxykinase
PGC-1α	Peroxisome proliferator-activated receptor gamma coactivator 1-alpha
P-gp	P-glycoprotein
PI3K/Akt	Phosphoinositide 3-kinase/protein kinase B
PIP2/PIP3	Phosphatidylinositol-4,5-bisphosphate/Phosphatidylinositol-3,4,5-Trisphosphate
PK/PD	Pharmacokinetic/pharmacodynamic
PLGA	Poly(lactic-co-glycolic acid)
PPAR-γ	Peroxisome proliferator-activated receptor gamma
PPG	Postprandial glucose
RCT	Randomised controlled trial
RES	Reticuloendothelial system
ROS	Reactive oxygen species
SGLT/SGLT1	Sodium-glucose co-transporter/Sodium-glucose co-transporter 1
SIRT1	Sirtuin-1
SLN	Solid lipid nanoparticle
SNEDDS	Self-nanoemulsifying drug delivery system
SOD	Superoxide dismutase
STZ	Streptozotocin
SULT	Sulfotransferase
T2DM	Type 2 diabetes mellitus
t½	Elimination half-life
TC	Total cholesterol
TG	Triglycerides
TNF-α	Tumour necrosis factor-alpha
TPP	Tripolyphosphate
UGT	UDP-glucuronosyltransferase
UPR	Unfolded protein response
US	United States
W/O/W	Water-in-oil-in-water
[IV]	In vitro
[PC]	Preclinical
[CC]	Clinical-conventional
[CN]	Clinical nano
[Epi]	Epidemiological
