# Supplementary material for: Harnessing Medicinal Plants Through Advanced Drug Delivery: A New Era in Type 2 Diabetes Management
Source: Pharmaceutics. 2026 Sep 1;18(9):1100. doi: 10.3390/pharmaceutics18091100 (PMC13610769; doi:10.3390/pharmaceutics18091100)
Supplement: Supplementary file 1 [file pharmaceutics-18-01100-s001.zip › pharmaceutics-4479261-supplementary.pdf]

Table S1. Comparative Scope Analysis of the Present Manuscript Against Representative Prior Reviews on Phytochemicals and Nanoformulation Strategies in Type 2 Diabetes Mellitus

**Feature**	**Dewanjee et al. 2020 [12]**	**Nie & Cooper 2021 [26]**	**Imam et al. 2021 [14]**	**Naz et al. 2023 [6]**	**Krawczyk et al. 2023 [7]**	**Present Manuscript**
Disease focus	Multiple metabolic diseases including T2DM, obesity, and NAFLD	T2DM and related metabolic disorders	Multiple diseases; not T2DM-specific	T2DM	T2DM with focus on inflammation and oxidative stress	T2DM across all mechanistic, translational, and regulatory axes
Phytochemicals addressed	Multiple compounds; general survey without systematic comparison	Multiple polyphenols; mechanism-focused	Multiple compounds; general survey	Polyphenols only	Polyphenols with emphasis on inflammatory pathways	Ten specific compounds with comparative mechanistic and clinical evidence
Nanocarrier platforms	General mention without platform-specific analysis	Not addressed	General survey without head-to-head comparison	Not addressed	Not addressed	Eight platforms compared across ten parameters including T2DM suitability, scalability, and regulatory status (Table 2A)
Clinical evidence grading	Not applied	Not applied	Not applied	Not applied	Not applied	GRADE framework applied to all phytochemicals with per-compound ratings (Table 5)
Evidence category separation	Preclinical and human data presented together without distinction	Partial separation	Not applied	Not applied	Not applied	Five separate evidence categories — human RCTs, conventional extract benchmarks, preclinical nanoformulation studies, epidemiological data, and evidence status summaries (Table 3)

Herb–drug interaction analysis	Not addressed	Not addressed	Not addressed	Not addressed	Not addressed	Structured pharmacokinetic interaction table covering CYP enzymes, drug transporters, and clinically documented interactions with verified evidence level (Table 4)
Regulatory pathway analysis	Brief mention	Not addressed	Not addressed	Not addressed	Not addressed	Comprehensive analysis of FDA, EMA, and CDSCO regulatory frameworks for nano-phytomedicine development (§7.2)
Gut microbiota mechanistic evidence	Not addressed	Partially addressed	Not addressed	Partially addressed	Not addressed	Dedicated sub-section covering SCFA signalling, *Akkermansia muciniphila*, bile acid metabolism, and TGR5/GLP-1 pathways (§6.2)
Epigenetic mechanisms	Not addressed	Not addressed	Not addressed	Not addressed	Not addressed	Dedicated coverage of DNMT inhibition, HDAC modulation, and miRNA regulation by phytochemicals (§6.2)
Nanocarrier platform comparison	Not provided	Not provided	Partial; no systematic head-to-head analysis	Not provided	Not provided	Structured head-to-head comparison table (Table 2A) covering eight platforms across ten parameters
Explicit literature search strategy	Not reported	Not reported	Not reported	Not reported	Not reported	Fully documented search strategy including databases, keywords,

						search period, inclusion and exclusion criteria, and quality assessment framework (§2.2)
Primary sources for clinical data	Not consistently provided	Partially provided	Not provided	Not provided	Not provided	All clinical evidence rows carry verified primary source citations with DOI and trial registry number where applicable
Search period	To 2020	To 2021	To 2022	To 2023	To 2023	January 2000 to August 2025
